# Extracellular Polymers from *Nitzschia* sp. for Removing Clay Minerals from Water in Mining

**DOI:** 10.3390/polym18101221

**Published:** 2026-05-17

**Authors:** Jeferson Grisales, Katiuska Huapaya, Gabriela Silva-Zamora, Luis A. Cisternas, Paris Lavin, David Jeison, Manuel Zapata, Mariella Rivas

**Affiliations:** 1 Laboratorio de Biotecnología Ambiental, Departamento Biotecnología, Facultad de Cs. del Mar y Recursos Biológicos, Universidad de Antofagasta, Avda. Angamos 601, Antofagasta 1230700, Chile; jeferson.grisales.giraldo@ua.cl (J.G.); katiuska.huapaya.mora@ua.cl (K.H.); gabriela.silva.zamora@ua.cl (G.S.-Z.); manuel.zapata@uantof.cl (M.Z.); 2 Programa Doctorado en Ciencias Aplicadas mención Sistemas Acuáticos, Facultad de Cs. del Mar y Recursos Biológicos, Universidad de Antofagasta, Avda. Angamos 601, Antofagasta 1230700, Chile; 3 Facultad de Ingeniería, Universidad de Antofagasta, Avda. Angamos 601, Antofagasta 1230700, Chile; luis.cisternas@uantof.cl; 4 Departamento de Biotecnología, Facultad de Cs. del Mar y Recursos Biológicos, Universidad de Antofagasta, Avda. Angamos 601, Antofagasta 1230700, Chile; paris.lavin@uantof.cl; 5 Escuela de Ingeniería Bioquímica, Pontificia Universidad Católica de Valparaíso, Avda. Brasil 2085, Valparaíso 2362803, Chile; david.jeison@pucv.cl

**Keywords:** kaolinite, *Nitzschia*, clays, recirculated water, flocculation

## Abstract

*Nitzschia* sp., a diatom isolated from Paposo (Antofagasta, northern Chile), was evaluated as a biological solution for removing kaolinite-type clay minerals from recycled process water in large-scale copper mining. Optimization of culture conditions to maximize extracellular polymeric substance (EPS) production revealed that supplementing with 0.1 gL^−1^ of glucose yielded the highest EPS levels on day 17, reaching 1285 ± 58.9 mgL^−1^ (control equal to 237.8 ± 34 mgL^−1^ on day 17). However, maximum dry weight biomass productivity was achieved in the presence of sodium carbonate at a concentration of 1 gL^−1^ (319 ± 12.5 mgL^−1^d^−1^), significantly exceeding the productivity of the control group (242.7 ± 5.4 mgL^−1^d^−1^). Notably, low glucose supplementation enhanced EPS synthesis. Application of control-derived EPS of 1 gL^−1^ rapidly decreased kaolinite initial turbidity from ~2024 FNU to ~354 ± 0.74 FNU within one minute. Even more glucose-derived EPS (1 gL^−1^) further reduced turbidity to ~22.2 ± 0.1 FNU at 5 min, achieving a flocculation efficiency of ~98.9% after 15 min. Genomic analysis and KEGG annotation identified abundant genes for EPS and carbohydrate metabolism, including numerous glycosyltransferases, glycoside hydrolases, and multiple copies of UDP-glucose 4-epimerase, consistent with strong polysaccharide-biosynthesis capacity. Physicochemical characterization (particle sizing, HPLC, SEM, zeta-potential and FT-IR) showed EPS comprised mainly of rhamnose, fucose, arabinose, xylose and glucose, featuring functional groups (–OH, C=O/COO–, O-acetyl, uronic/guluronic signatures) that interact with kaolinite to promote aggregation. These findings demonstrate that *Nitzschia*-derived EPS, especially from glucose-supplemented cultures, represent promising sustainable bioflocculants for treating kaolinite-contaminated recycled water in mining operations.

## 1. Introduction

The Chilean copper-mining industry is largely situated in arid regions where water is naturally scarce, and its use in mineral processing has significant social and environmental impacts [1]. To mitigate this, the industry has focused on water recirculation. Mining operations rely on three water sources: continental water (19%, 12.09 m^3^s^−1^), ocean water (8%, 5.28 m^3^s^−1^) and, most critically, recirculated water from the mining processes themselves (73%, 46.98 m^3^s^−1^) [2]. This recirculated water comprises all flows re-injected into the process, whether treated or not [3]. In the face of accelerating global climate change, enhancing the efficiency of water use by increasing both the quantity and quality of recovered water is not just an operational goal; it is an environmental and economic imperative.

A major obstacle to recovering high-quality recirculated water from thickeners and tailings for reuse in mineral flotation is the presence of complex clay minerals [4]. These are not incidental impurities; clays are an intrinsic part of the gangue materials (waste rock) in numerous copper, platinum, and nickel deposits [4,5,6]. Clays are phyllosilicates characterized by anisotropic structures and colloidal particle sizes. This fine nature in kaolinite, for example, allows them to form highly stable colloidal suspensions in water unless the particles are large [7], which is not the case in mineral processing residues. These suspended clay colloids interfere with mineral flotation—a key process for separating valuable minerals from waste—thereby reducing recovery rates. Then, industry has developed various strategies to mitigate their negative effects, including rheology modifiers or reagents that adsorb to clay surfaces to prevent adhesion to valuable minerals [4,8,9,10]. However, removing these clays from the water circuit remains a primary challenge.

To accelerate the removal of suspended particles, the industry widely employs polymers known as flocculants [10,11,12]. These long-chain polymers, such as synthetic polyacrylamide (PAM), metal salts, alum or ferric chloride [13], function by bridging multiple particles into larger aggregates, or “flocs”, which then settle more rapidly under gravity [14,15]. However, while these flocculants accelerate the settling rate, they raise water ionic strength, necessitating further treatment such as adsorption or membrane filtration. Moreover, PAM can degrade into toxic monomers, which are harmful to both the environment and human health. This has led to restrictions on their use in many countries, pushing the industry to find safer, more sustainable alternatives [16,17,18,19].

In this study, we propose a renewable, environmentally friendly bioflocculant based on extracellular polymeric substances (EPS) produced by the diatom *Nitzschia* sp. to remove clay minerals from recirculated mining water. Diatoms are major eukaryotic phytoplankton, responsible for approximately 40% of marine primary productivity and 25% of global primary productivity [20]. They play key roles in the global silicon [21], carbon, nitrogen, and iron cycles [22]. For instance, EPS secreted by diatoms such as *N. curvilineata* serve as labile organic carbon sources [23,24,25] and help stabilize marine sediments by lowering erosion rates [26,27].

Diatoms secrete substantial amounts of EPS, a complex mixture of polysaccharides, proteins, lipids, and nucleic acids [28,29]. In nature, these substances serve multiple functions: they promote attachment to surface, aid in motility, and are central to biofilm formation. Critically, they are known to stabilize marine sediments and facilitate their deposition through specific binding to clay particles [16,27,28,30].

The mechanisms behind this natural flocculation involve multiple factors. (i) Direct binding: The chemical composition of EPS is key. Their negatively charged groups (e.g., carboxyls) can act as ligands, binding to metal ions via ion exchange mechanisms, while amino groups can facilitate electrostatic interactions with particles [31,32]. This allows EPS to effectively “glue” sediment grains together [33]. (ii) Drag reduction: the polymeric nature of EPS can alter the hydrodynamics around particles, reducing drag and promoting settling [34,35]. (iii) Particle trapping: diatoms can form reinforced EPS networks, such as tubular structures, that physically trap particles, enhancing floc formation and stability [28,32]. Recent work has highlighted these EPS–clay flocculation processes in coastal marine environments, demonstrating their ability to enhance both the sedimentation rate and the stability of the resulting flocs [32].

On the other hand, EPS production and composition also respond to environmental conditions, nutrient availability, or in a species-specific manner, rev. in [36]. For example, stress conditions (e.g., nitrogen or phosphorus starvation) can increase triacylglycerol (TAG) biosynthesis and the accumulation of high-value molecules, including EPS [37]. This approach offers a feasible method for enhancing the yield of the bioflocculant utilized in this study, in which diverse nutrient and carbon sources were employed to assess the impact on the growth of *Nitzschia* sp. and their effect on growth, EPS production, EPS functional groups, and their capacity to form flocs and accelerated sedimentation rates in the presence of clays.

Therefore, to gain a deeper understanding of the biological processes involved, we also identified genes related to EPS metabolism by sequencing the genome of *Nitzschia* sp. strain 53.3, isolated from the Antofagasta coast. This genomic analysis enabled the identification of specific genes and metabolic pathways involved in EPS synthesis, providing a scientific foundation for future optimization. Finally, we demonstrated the effectiveness of synthesized EPS by evaluating its sorption properties and its capacity to promote the flocculation and sedimentation of clay minerals, confirming its potential as a viable alternative to conventional flocculants.

## 2. Materials and Methods

### 2.1. Isolation, Selection, and Controlled Cultivation of Microalgae

Environmental samples from sediments and water column in the Paposo were collected in June 2014. Original samples collected were enriched with the following culture media F/2 medium (882 μM NaNO_3_, 36.2 μM NaH_2_PO_4_, 106 μM Na_2_SiO_3_ × 9H_2_O, 11.7 µM FeCl_3_ × 6H_2_O, 11.7 μM Na_2_EDTA × 2H_2_O, 39.3 nM CuSO_4_ × 5H_2_O, 26 nM Na_2_MoO_4_ × 2H_2_O, 76.5 nM ZnSO_4_ × 7H_2_O, 42 nM CoCl_2_ × 6H_2_O, 0.91 µM MnCl_2_ × 4H_2_O, 0.29 µM thiamine HCl, 2.05 nM biotin, 0.36 nM cyanocobalamin) using sea water as the matrix adjusted to pH 6.5 and autoclaved in Erlenmeyer flasks. Microalgae isolated by microfishing [38] were serially diluted and streaked on agar plates to obtain single isolated cells. They were selected based on robust and fast growth in the specific culture medium. Then, unialgal cultures were scaled subsequently at 2, 10, 50, 100, and 1000 mL. Finally, monospecific cultures were scaled up to a larger volume in 20 L bottles to obtain biomass by bubbling air filtered to 0.22 µm through a lift aeration system. The control culture conditions were 24 °C with continuous illumination at 100 μmol photons m^−2^·s^−1^ with cold-white fluorescent lamps (36 Watts). All light intensities were measured with a quantum sensor (LI-250A, LI-COR Biosciences, Lincoln, NE, USA). The culture was maintained for 26 days without pH control. The cells were analyzed using an Olympus BX43 epifluorescence microscope. At the end of the growth period, biomass was harvested and lyophilized, resulting in algal powder for use in biochemical and molecular characterization and maintained at −20 °C until use.

### 2.2. Extraction of Total DNA, Amplification of 18S rDNA, Phylogenetic Analysis and Sequencing

Identification of microalgae sample was carried out by extracting genomic DNA using a PowerSoil pro DNA Isolation kit (Qiagen N.V., Hilden, Germany) according to the manufacturer’s procedure and using the following primers, 18S-C and 18S-D, for ribosomal gene 18S rDNA [39]. DNA amplification was carried out using Sapphire Fast PCR Master Mix (Takara) in a Thermal Cycler AB2720 (Applied Biosystems) under the following conditions: initial denaturation at 95 °C for 30 s; 35 cycles of 95 °C for 30 s, annealing temperatures corresponding to 67 °C during 1 min and 72 °C for 1 min; and final extension at 72 °C for 5 min. PCR products were purified using a DNA purification kit (Promega) and sequenced by Macrogen Inc. (Korea). The sequences were edited and assembled using Chromas Pro 1.5 software and subsequently analyzed using CLC Main Workbench (version 6.7.1; Qiagen) and BlastN software v.2.17.0 against the nr database available in GenBank (http://blast.ncbi.nlm.nih.gov/Blast.cgi ) with a cutoff of 1 × 10^−5^. Genome sequencing was performed at NOVOGENE Inc. using the NovaSeq PE150 Illumina platform. The raw data are transformed into Sequenced Reads or raw reads by base calling. Raw data are recorded in a Fastq file. In silico analysis was performed using software such as KAAS-KEGG Automatic Annotation Server (https://www.genome.jp/kegg/kaas/), KBase (https://www.kbase.us/), HMMER v3.4 (http://hmmer.org/), Blast+ v.17.0, and Geneious Prime v2025.2.2 (Dotmatics, UK).

### 2.3. Growth Measurements

For growth curve experiments, cells growing in controlled culture conditions were harvested and resuspended in fresh medium at an initial biomass concentration of ~10^6^ cells mL^−1^ in 1 L bottles. Density was measured with a Neubauer chamber (Marienfeld, Germany) using an Olympus BX43 microscope. At the end of the growth period, the biomass was harvested and lyophilized, resulting in algal powder for use in biochemical and molecular characterizations and maintained at −20 °C until use. Dry biomass was determined as described by Khaw et al. [40]. Particularly, for dry biomass determination of saline samples in seawater, aliquots (5 mL) taken from cells in culture were washed with ammonium formate previously to remove excess salts. Specific growth rates of cultures were calculated using the expression:

(1) $$µ\;=\;\ln\left(C/C_{0}\right)/t$$

where µ is the specific growth rate, *C*_0_ is the initial biomass concentration (dry biomass) and *C* is the biomass concentration at any time, *t*. Biomass productivities were calculated from dry biomass data according to the expression:

(2) $$P\;=\;\left(C\;-\;C_{0}\right)/t$$

where *P* is the specific productivity. Alternatively, the absorbance at 680 nm was measured to obtain information about the cell chlorophyll content [41].

### 2.4. Biochemical Characterization of Microalgae

Chlorophylls and carotenoids were measured spectrophotometrically using pigment extracts from 10 mg algal powder. The powder was mixed with 5 mL pure methanol, incubated for 8 min at 60 °C, sonicated for 8 min and centrifuged for 10 min at 1814× *g*, 4 °C. The supernatant was incubated in the dark at −20 °C. The extraction was repeated three times to give a pellet without green color. Finally, 1 mL pigment extract was mixed with 4 mL pure methanol and shaken vigorously prior to measurement. Then, pigment contents in the supernatant were determined by photometry as absorbance at 480, 630 and 664 nm, respectively. Pigment concentrations were calculated as follows:

Chl *a* (μg mL^−1^) = 13.2654(A664) − 2.6839(A630), (3)

Chl *c* (μg mL^−1^) = 28.8191(A630) − 6.0138(A664), (4)

Carotenoids (μg mL^−1^) = 10(A480) (5)

The Lowry method [42] was used to measure the protein content of pretreated biomass as described. Aliquots containing 10 mg of freeze-dried biomass were pretreated by milling for 20 min in 5 mL X-lysis buffer (50 mM KH_2_PO_4_, pH 7.8; 0.1 mM EDTA-Na; 1% [*v*/*v*] Triton X-100; 0.01% Protease Inhibitor Cocktail [Sigma]) and 100 mg of alumina in a vortex to facilitate protein extraction [43]. Samples were processed in the dark to prevent degradation of the Folin–Ciocalteau reagent (cat. n° BM-1490, Winkler, Chile). The absorbance of triplicate samples at 750 nm was measured on a UV-vis spectrophotometer (MultiScan GO, Thermo Scientific), and absorbance values were converted to protein concentrations using a calibration curve established with bovine serum albumin. Colorimetric total carbohydrate analysis was performed according to Dubois et al. [44] using a series of glucose solutions as the standard (0–100 mgL^−1^). The algal extract used for carbohydrate measurement was subjected to analytical acid hydrolysis as method of cell rupture. It was carried out with 10 mg of dry biomass in 5 mL 2.5 M HCl (Spectroquant TR 320 Thermocycler, Merck, Germany). This mixture was neutralized with 5 mL 2.5 M NaOH to finally obtain the algal extract. Aliquots of 50 µL algal extract were mixed with 450 µL deionized water, 500 µL phenol solution (5% *w*/*v*) and 2.5 mL concentrated sulfuric acid. The reaction was incubated for 10 min at room temperature and then for 30 min in a 35 °C water bath with vortexing every 5 min. Absorbance of samples at 483 nm was measured on the UV-vis spectrophotometer.

### 2.5. Determination of Culture Viscosity over Time

One liter of *Nitzschia* sp. strain 53.3 was cultured, and 50 mL samples were collected in the lag, log and stationary phases (days 0, 3, 6, 7, 10 and 16). The apparent viscosity of the microalgae suspension was then determined at 25 °C using an Anton Paar model MCR 102 rheometer. Rheological measurements were performed using a shear rate of 20 to 350 s^−1^. All measurements were performed in triplicate, and the averages were reported with their standard deviation.

### 2.6. Extraction, Characterization, and Identification of EPS

For EPS extraction, the protocol described by Liu et al. [45] and Bafana [46] was followed. Briefly, the cultures were centrifuged at 4500× *g* for 20 min at 4 °C to recover the supernatant. The supernatant was incubated at 60 °C with agitation for 16 h, and then precipitated with methanol in a 1:1 (*v*:*v*) ratio. Finally, the mixture was incubated at 4 °C for 16 h to obtain the EPS precipitates. The precipitated EPS were resuspended in Milli-Q water, and the precipitation and redissolution process was repeated twice to improve purification [29]. Then, the wet weight of EPS was determined in mg. Finally, the samples were stored at −80 °C and then lyophilized to white powder using a freeze dryer (FreeZone 1, Labconco, USA) [47]. After freeze-drying, dry weight was determined using an analytical balance to calculate mgL^−1^.

EPS productivity was determined in the log phase and stationary phase (day 7 and day 17, respectively) using the following formula:

(6) $$Productivity\;\left(\frac{g}{Ld}\right)=\frac{Dry\;weight}{t1-t0}$$

### 2.7. Monosaccharide Composition

Monosaccharide composition of polysaccharide was evaluated by High-Performance Liquid Chromatography (HPLC) coupled to a diode array detector (HPLC-DAD) following acid hydrolysis and pre-column derivatization with 1-phenyl-3-methyl-5-pyrazolone (PMP), as previously described [48]. Briefly, 2 mg of EPS were hydrolyzed with 2 M trifluoroacetic acid at 120 °C for 2 h in sealed vials. The acid was removed under reduced pressure, and residues were co-evaporated twice with methanol before reconstitution in ultrapure water. For derivatization, 50 µL of hydrolysate was mixed with 0.3 M NaOH and 0.5 M PMP in methanol and incubated at 70 °C for 30 min. The reaction was neutralized with 0.3 M HCl, and excess PMP was removed by chloroform extraction. Samples were filtered (0.22 µm) prior to analysis. Separation was performed on a Mediterranea Sea18 column (250 × 4.6 mm, 5 µm; Teknokroma, Spain) at 30 °C using 10 mM ammonium formate (solvent A) and acetonitrile (solvent B) under a linear gradient from 15% to 23% B. The flow rate was 1.0 mLmin^−1^ and detection was conducted at 245 nm [49]. The constituent monosaccharides were identified by determining their elution time compared to the standards (L-Rha, L-Fuc, L-Ara, D-Xyl, D-Man, D-Gal, D-Glc). The quantification of the monosaccharides was then confirmed by injecting different concentrations of monosaccharide standards and plotting the response area as a function of concentration.

### 2.8. Assays for Optimization of EPS

To optimize EPS production in *Nitzschia* sp., different physicochemical growth parameters were evaluated using variable nutrient concentrations, considering a targeted one-factor-at-a-time (OFAT) approach including five or six levels, as detailed in Table 1. F/2 supplemented with glucose or sodium carbonate (Na_2_CO_3_) was used, and in the case of sodium metasilicate (Na_2_SiO_3_), the concentrations were adjusted according to Table 1. F/2 medium in SW was used as a control. Each condition was inoculated with 1.3 × 10^6^ cells mL^−1^. All cultures were performed in triplicate. Optical density was determined at 680 nm in the lag, log, and stationary phases (days 0, 3, 6, 7, 10, and 17). To determine EPS productivity, samples were taken in the log and stationary phases (day 7 and 17, respectively).

The cultures were maintained for 17 days at 24 °C with continuous illumination at 100 μmol photons m^−2^·s^−1^, without pH control, and counts performed every 3 days in a Olympus BX43 epifluorescence microscope.

### 2.9. Analysis Using Fourier Transform Infrared Spectroscopy (FT-IR)

The extracted EPS were analyzed using FT-IR according to Lilo et al. [50] to identify differences in EPS composition synthesized by *Nitzschia* sp. cultivated under various experimental conditions. Briefly, 10 mg of lyophilized EPS was used in a JASCO FT/IR-4600 device within the spectrochemical range composed of absorption intensities for each wavelength number of the mid-infrared spectrum (4000–400 cm^−1^). SpectraManager 2.5 software (JASCO) was used to process spectral data.

### 2.10. Zeta Potential Analysis

The surface charges of EPS, kaolinite, and their interactions were determined by measuring the zeta potential (ς) using a Litesizer DLS 500 (Anton Paar). About 0.1 g of each sample was diluted in 10 mL of DI water and 1 mL of the diluted sample was injected into a flow cell. The measurements were performed by adjusting the pH to 2, 4, 6, 8, 10, and 12 with 0.1 M NaOH or HCl solutions. All values were measured in triplicate at a temperature of 25 °C.

### 2.11. Clay Flocculation and Sedimentation Tests

Kaolinite (Ward Science, Inc.) previously characterized by XRD, Zeta potential, and FT-IR was used for the tests. Flocculation was performed through sedimentation tests using EPS as a biological flocculant interacting with kaolinite. The protocol described by Ho et al. [51] and Huapaya et al. [52] was followed with modifications. Deionized water at pH 8.0 was used for the tests in a final volume of 300 mL. Kaolinite of 1 gL^−1^ was added and homogenized at 300 rpm for 10 min. EPS of each treatment was then added at concentrations of 0.1 gL^−1^ and 1 gL^−1^ and homogenized at 100 rpm for 10 min. Subsequently, supernatant turbidity after flocculation was determined at 0, 5, 10, 15, 30, and 60 min using a Hanna turbidimeter (HI98713-02) in Formazin Nephelometric Units (FNU). Water clarification was compared to negative control with kaolinite of 1 gL^−1^ without EPS. The commercial chemical flocculant SNF604 0.1 gL^−1^ was used as a positive control. Each treatment was performed in triplicate. The sedimented flocs were kept at −80 °C and subsequently freeze-dried in a freeze dryer at −55 °C (Operon, Korea) for further analysis. In parallel, flocculation efficiency (FE) was calculated as follows:

(7) $$FE\left(\%\right)=\;\frac{{FNU}_{control\;}-\;{FNU}_{test}}{{FNU}_{control}}\;\times 100$$

where FNU_control_ corresponds to the turbidity value of the experiment under control conditions (kaolinite without flocculant), and FNU_test_ is the turbidity value of test experiment (with EPS and SNF604).

### 2.12. Scanning Electron Microscopy (SEM)

The EPS and flocs obtained were visualized using scanning electron microscopy (SIGMA 300 VP, ZEISS), equipped with X/EDS electrons, at an acceleration voltage of 30 kV. The samples were deposited directly onto carbon adhesive tape and then gold-plated for visualization.

### 2.13. Statistical Analysis

Unless otherwise indicated, all data represents means of three independent experiments, and the standard error was calculated from three replicates. Growth rate, biomass productivity, biochemical composition, and EPS productivity data for microalgal strains were analyzed using a one-way analysis of variance. Statistical analyses were performed with statistical significance assessed at a 95% confidence level (*p* < 0.05).

## 3. Results

### 3.1. Identification and Characterization of Nitzschia sp. Strain 53.3 and Its EPS

**Figure 1:**
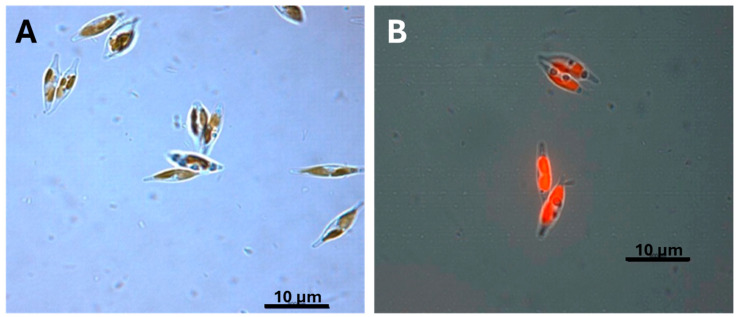
Optical microscopy in *Nitzschia* sp. strain 53.3. (**A**) Clear field microscopy; (**B**) epifluorescence microscopy highlighting the autofluorescence of chlorophyll.

The diatom strain was amplified until a pure culture was obtained, and photographs of the strain were taken using an epifluorescence microscope at 400× and 1000× magnification, which allowed for preliminary identification of the microalgae (Figure 1). The morphology presented is consistent with a diatom of the genus *Nitzschia*, with cells measuring between 10 and 12 µm in length and 3 and 6 µm in width. Overall, the cells are oval and elongated at both ends (Figure 1A), and two large central chloroplasts are visible (Figure 1B).

Molecular phylogenetic analysis was used to further determine their taxonomic position. 18S rDNA gene partial sequence consisting of 1678 nucleotides (nt) was determined and submitted to GenBank (accession nos. PZ094155), and BlastN was applied to find regions of similarity. Phylogenetic analysis of 18S rDNA gene sequences demonstrated that our isolated strain belongs to the algal class Bacillariophyceae. The strains of *Nitzschia* sp. strain 53.3 share 98.81% identity with *Nitzschia closterium* strain MACC-B228 (EF553459.l), with *e*-value equal to 0.0.

**Figure 2:**
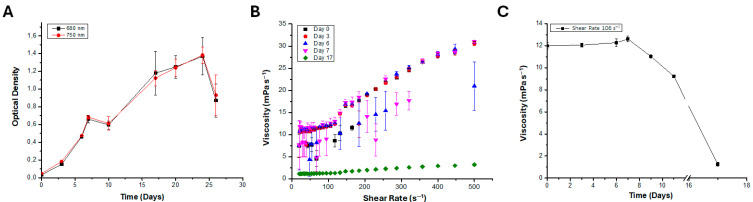
The growth of *Nitzschia* sp. strain 53.3 was measured. (**A**) Optical density of the culture between days 2 and 26; (**B**) viscosity of the culture according to growth phases between days 0 and 17; (**C**) viscosity of the culture in mPa·s at a shear rate of 106 s^−1^. Bars indicate standard error of triplicate experiments.

As illustrated in Figure 2, the growth curve was determined from optical density at 680 and 750 nm. This analysis included observations of latency, lag, and stationary phases (Figure 2A). The diatom cultivated at 24 °C reaches a maximum optical density at 17 days equal to OD_680nm_ of 1.18 (equal to 24.5 × 10^6^ cell mL^−1^), and remains in the stationary phase for approximately 8 days. Also, temporal progression of the viscosity of the Nitzschia sp. culture was ascertained through systematic measurement at varying shear rates from 0 to 500 s^−1^ over a period of 17 days (Figure 2B). The data demonstrated a marked increase in viscosity up to day 7, followed by a precipitous decline on day 17. By establishing a shear rate of 106 s^−1^ during the 17 days of cultivation, it was determined that the culture has low viscosity (equal to 11.983 mPa s^−1^), which increases progressively until day 7 (corresponding to 12.611 mPa s^−1^) and finally dramatically decreases to 1.247 mPa s^−1^ on day 17 (Figure 2C).

Additionally, it was determined that the biomass of *Nitzschia* sp. strain 53.3 harvested at stationary phase (Day 17) had a protein content corresponding to 12.37 ± 0.009% g g^−1^ dry weight (dw) biomass, lipids corresponding to 21.66 ± 0.065% g g^−1^ dw biomass, 9.99 ± 0.011% carbohydrates g g^−1^ dw biomass and 3.99 ± 0.14 mgGAE g^−1^ dw total phenolic compounds. Pigment analysis yielded 171.27 ± 28.02 µg mL^−1^ total chlorophylls and 87.15 ± 8.47 µg mL^−1^ total carotenoids, while HPLC quantification against a standard indicated fucoxanthin production of 7.83 mg g^−1^ dw biomass. Biochemical characterization of the EPS revealed no detectable proteins or nucleic acids, and polysaccharides represent between 12 and 15% g g^−1^ dw biomass and were composed of rhamnose, fucose, arabinose, xylose, and glucose.

**Figure 3:**
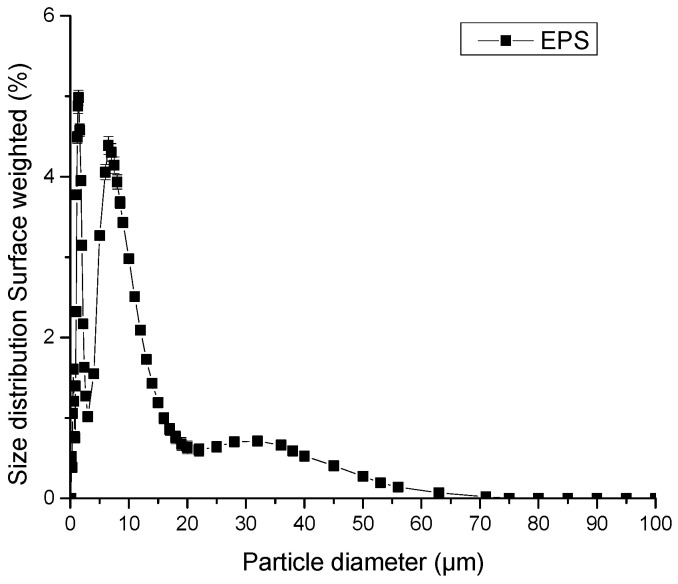
Particle size distribution for EPS extracted from cultures of *Nitzschia* sp. strain 53.3. Bars indicate standard error of ten experiments.

A subsequent analysis of the particle size distribution of EPS revealed a distribution below 60 µm, with the highest percentage found in the 2-to-10 µm size range (Figure 3).

**Figure 4:**
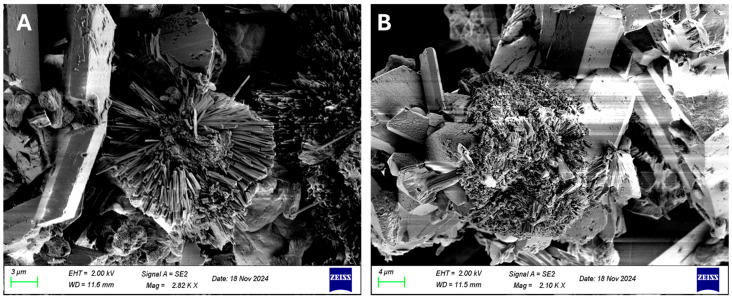
SEM showing the microscopic characteristics of EPS from *Nitzschia* sp. strain 53.3 with magnification (**A**) 1.24 and (**B**) 1.20 K X.

The EPS isolated from *Nitzschia* sp. were examined by SEM to assess their three-dimensional structure. Surface morphology revealed regularly patterned structures, including hexagonal motifs, alongside amorphous crystalline layers with an average size of 10 µm (Figure 4), consistent with the particle size previously determined (Figure 3). Such EPS architecture may increase the available contact surface, facilitating interactions with other molecules.

The *Nitzschia* sp. strain 53.3 genome was sequenced using the Hiseq Illumina platform, yielding high-quality sequences with Q20 equal to 97.28% and Q30 greater than 92.55% of the sequences, with a GC content of 51.82%. Subsequently, raw reads containing adapters (387,294 reads, 0.51%), reads containing N > 10% (3998 reads, 0.01%), and low-quality reads (1692 reads, 0.00%; Qscore ≤5) were removed. After the cleaning process, the clean reads equaled 75,808,242 reads (99.48%) with 11.4 Gb. Bioinformatic analysis was performed using Geneious Prime^®^ 2025.2.2, KBase software and Kyoto Encyclopedia of Genes and Genomes (KEGG) platforms for genes related to carbon metabolism and exopolysaccharides. According to KEGG, the presence of genes related to the citrate cycle (*n* = 306), glycolysis (*n* = 248), glycosyltransferases (*n* = 195), glycoside hydrolases (*n* = 108), and others was determined (Supplementary Data, Table S1).

### 3.2. Growth and Production of EPS Under Different Growth Conditions

To determine the influence of various factors on EPS production, a multivariate approach was initially implemented using a three-level Box–Behnken design, considering pH, sodium carbonate, and sodium metasilicate as key factors. This design allowed the evaluation of both main effects and interaction terms within the selected experimental space. The statistical analysis (performed in Minitab 17) showed that the model was significant for biomass production (*p*-value = 0.006; R^2^ = 95.75%; adjusted R^2^ = 88.09%), indicating a good fit. However, for EPS production, the model did not reach statistical significance (*p*-value = 0.087). Although the R^2^ value (86.57%) suggests that a large portion of variability was explained, the adjusted R^2^ (62.40%) and especially the predicted R^2^ (0.00%) indicate limited robustness and poor predictive capability. Response surface plots (Supplementary Data, Tables S2 and S3, Figures S1 and S2) further illustrate that, although interactions were formally evaluated, they did not show consistent or interpretable effects on EPS production within the tested experimental domain. This outcome likely reflects the intrinsic biological variability of EPS biosynthesis, as well as the possibility that relevant nonlinear or interaction effects occur outside the selected factor ranges. Consequently, while the DOE framework provided useful exploratory insight, it did not yield a reliable predictive model for EPS optimization.

**Figure 5:**
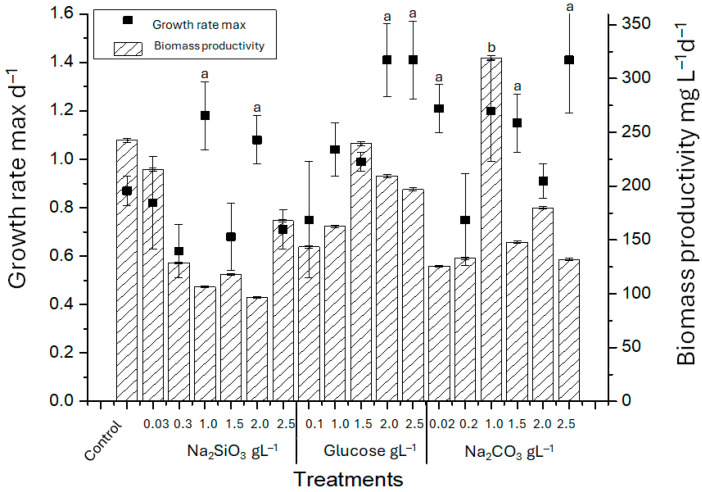
Growth rate for *Nitzschia* sp. under different culture conditions and daily biomass productivity in mg L^−1^d^−1^. a corresponds to statistically significant differences compared to the control group on growth rate max; b corresponds to statistically significant differences compared to the control group on biomass productivity. Bars indicate standard error of triplicate experiments.

Based on these observations, a one-factor-at-a-time (OFAT) approach was subsequently employed as a targeted screening strategy to identify nutrient conditions that enhance EPS productivity. This approach examines different concentrations of key nutrients, such as carbon and silica sources, and their impact on growth and EPS production in *Nitzschia* sp. strain 53.3. Maximum growth rates and daily productivity were determined under culture conditions with varying concentrations of carbon and silicate (Figure 5).

When growth rate max (d^−1^) was measured for *Nitzschia* sp. cultures supplemented with varying concentrations of sodium metasilicate (Na_2_SiO_3_), glucose (C_6_H_12_O_6_), and sodium carbonate (Na_2_CO_3_), values varied relative to the control (0.8740 ± 0.06 d^−1^). The highest growth rates were observed with sodium metasilicate at 1 and 2 g·L^−1^ (1.1806 ± 0.14 and 1.0813 ± 0.1 d^−1^, respectively), glucose at 2 and 2.5 g·L^−1^ (1.4124 ± 0.15 and 1.4065 ± 0.16 d^−1^, respectively), and 2.5 g·L^−1^ sodium carbonate (1.4050 ± 0.22 d^−1^). Biomass productivity also differed significantly from the control group (242.66 ± 5.4 mg·L^−1^d^−1^). The maximum productivity (319.17 ± 12.5 mgL^−1^d^−1^) was obtained with 1 gL^−1^ sodium carbonate. Productivity levels similar to the control were observed with 0.03 g·L^−1^ sodium metasilicate (215.3 ± 22.5 mgL^−1^d^−1^), and glucose at 1.5 g·L^−1^ and 2 g·L^−1^ (239.5 ± 6.11, and 209 ± 5.61 mg·L^−1^d^−1^, respectively) (Figure 5).

**Figure 6:**
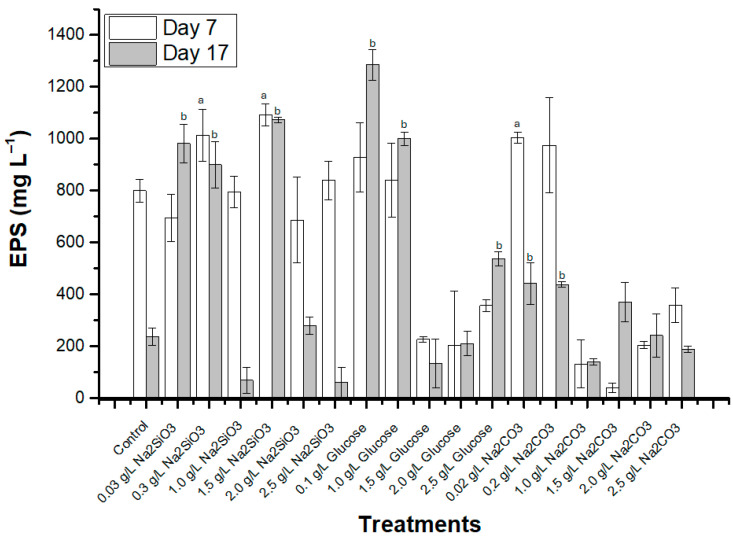
EPS production (mgL^−1^) in *Nitzschia* sp. strain 53.3 cultivated in different concentrations and nutrients on days 7 and 17 of cultivation. a corresponds to statistically significant differences compared to the control group on day 7; b corresponds to statistically significant differences compared to the control group on day 17. Bars indicate standard error of triplicate experiments.

EPS produced under different growth conditions was quantified in both the exponential and stationary phases (days 7 and 17), and EPS yield relative to cell biomass exhibited marked variability in response to nutrient concentrations (Figure 6).

In the log phase (day 7), cultures supplemented with 1.5 gL^−1^ sodium metasilicate yield the highest EPS concentration relative to the control condition (800 ± 43.7 mgL^−1^ EPS), reaching 1091.7 ± 41.6 mgL^−1^ EPS (Figure 6). In contrast, by the lag phase (day 17), EPS production declined markedly in control (237.8 ± 34 mgL^−1^ EPS) and in most treatments, except for 0.1 gL^−1^ glucose (1285 ± 58.9 mgL^−1^ EPS) and 1.5 gL^−1^ sodium metasilicate (1073.3 ± 10.4 mgL^−1^ EPS.

EPS productivity (Supplementary Data, Table S4) in the log phase (day 7) was as follows. The control condition presented a value of 114.3 ± 4.37 mgL^−1^d^−1^. The cultures treated with 1.5 gL^−1^ sodium metasilicate had highest EPS productivities of 156 ± 4.16 mgL^−1^d^−1^. In cultures supplemented with 0.1 gL^−1^ glucose, the determined major EPS productivity corresponded to 132.6 ± 3.38 mgL^−1^d^−1^. Finally, for *Nitzschia* sp. cultivated using 0.02 gL^−1^ sodium carbonate, the highest EPS productivity value determined was 143 ± 2.23 mgL^−1^d^−1^. Furthermore, EPS productivity in the lag phase (day 17) in *Nitzschia* sp. cultures was also determined and found to decline markedly relative to day 7, with responses varying according to nutrient concentration (Supplementary Data, Table S4). Based on EPS productivity, two nutrient treatments (1.5 gL^−1^ sodium metasilicate and 0.1 gL^−1^ glucose) and the control condition were selected for subsequent analyses.

### 3.3. Analysis of Zeta Potential of EPS

**Figure 7:**
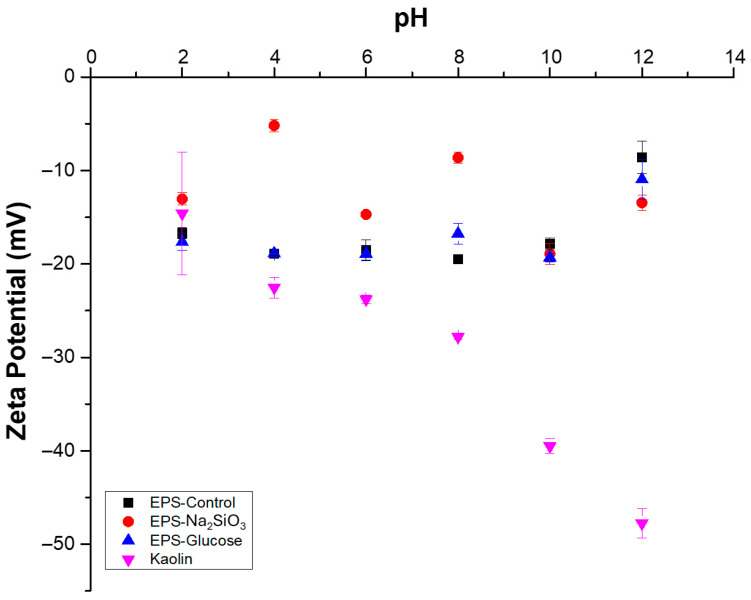
Zeta potential in deionized water of EPS in the pH range 2.0 to 12 from *Nitzschia* sp. cultivated in sodium metasilicate, glucose, and control conditions. Bars indicate standard error of triplicate experiments.

Surface properties of particles critically determine colloidal stability, and zeta potential magnitude is a key indicator of that stability. Zeta potentials of each EPS type were measured under identical pH, temperature and concentration conditions (of 0.1 gL^−1^). EPS from *Nitzschia* sp. exhibited zeta potentials between −5 and −20 mV across pH 2.0–12 in deionized water (Figure 7), indicating instability and a propensity for aggregation and flocculation. The control EPS samples at pH 2.0 had a zeta potential of −16.7 ± 0.57 mV; at pH 8.0, the zeta potential decreased to −19.48 *±* 0.47 mV and reached its highest value at pH 12, at −8.59 ± 1.75 mV. EPS from cultures supplemented with 0.1 g·L^−1^ glucose displayed a trend from pH 2.0 to 10, with zeta potential of −17.64 ± 0.91 mV at pH 2.0. Next, at pH 8.0, the value was −16.75 ± 1.09 mV, with the highest value occurring at pH 12, where the zeta potential was −10.95 ± 2.11 mV. EPS from cultures treated with 0.03 g·L^−1^ sodium metasilicate did not show a clear trend, with values varying across the pH range; for example, at pH 2.0, the zeta potential was −13.04 ± 0.65 mV, at pH 4.0, the value was −5.19 ± 0.66 mV, and at pH 8.0, the value was −8.63 ± 0.58 mV. By contrast, stable suspensions typically exhibit an absolute zeta potential greater than ±30 mV, as observed for kaolinite at pH 8.0 with a value of −27.8 ± 0.24 mV, and then at pH 10 and 12 with values of −39.5 ± 0.8 mV and −47.8 ± 1.58 mV, respectively (Figure 7).

### 3.4. Sedimentation and Flocculation Tests of EPS with Clays

**Figure 8:**
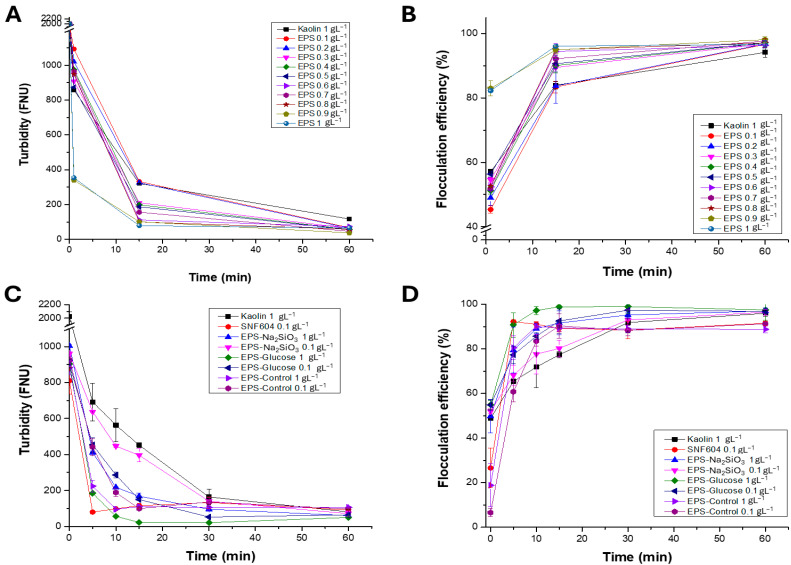
Sedimentation and flocculation tests for kaolinite and EPS from *Nitzschia* sp. cultivated under different conditions at pH 8.0. (**A**) Sedimentation curves in the presence of EPS from control condition at 0.1 to 1 gL^−1^; (**B**) flocculation efficiency determined for EPS from control condition from 0.1 to 1 gL^−1^; (**C**) sedimentation curves in the presence of EPS from different treatments at 0.1 and 1 gL^−1^; (**D**) flocculation efficiency for EPS from the different treatments. SNF604, chemical flocculant control. Bars indicate standard error of triplicate experiments.

Sedimentation tests were performed with kaolinite of 1.0 gL^−1^ and EPS concentrations ranging from 0.1 to 1.0 gL^−1^. Turbidity was measured at 1, 15, and 60 min. The initial turbidity of kaolinite (~1500 to 2000 ± 4.6 FNU at time 0) decreases to 340.3 ± 1.97 and 354 ± 0.74 FNU in the presence of 0.9 and 1 gL^−1^ EPS, respectively (Figure 8A), whereas the kaolinite control retained high turbidity, decreasing only to 321 ± 0.27 FNU at 15 min. The highest flocculation efficiency at 1 min was observed at 0.9–1.0 gL^−1^ EPS, reaching 82.9%. At 15 min, turbidity decreased markedly, with optimal flocculation efficiencies of 95%, 94.9%, and 96% for EPS concentrations of 0.8, 0.9, and 1 gL^−1^, respectively (Figure 8B).

Subsequent sedimentation assays employed kaolinite (1.0 gL^−1^) with EPS produced under different *Nitzschia* sp. strain 53.3 culture conditions (control, 0.03 gL^−1^ sodium metasilicate, and 0.1 gL^−1^ glucose), and chemical flocculant 0.1 gL^−1^ SNF604 as a positive flocculation control. Results showed substantial variation in turbidity reduction among the EPS samples (Figure 8C). Clay mineral kaolinite alone exhibited only a gradual turbidity decline from ~2000 FNU to 452.7 ± 12.42 FNU at 15 min, indicating limited natural sedimentation. Chemical flocculant SNF604 (0.1 gL^−1^) produced a pronounced turbidity reduction to 115.5 ± 13.5 FNU, demonstrating high flocculation efficiency.

EPS treatments also effectively induced flocculation. EPS in control condition at 0.1 and 1 gL^−1^ started at 925.5 ± 18.5 and 883 ± 40.5 FNU, respectively, and fell to 99 ± 5.5 and 109 ± 5.1 FNU after 15 min. In the context of utilizing EPS from *Nitzschia* sp. cultivated in the presence of sodium metasilicate at concentration of 0.1 and 1 gL^−1^, the initial turbidity levels correspond to 961 ± 11.5 and 1005 ± 5 FNU, respectively, and decrease to 396.5 ± 37.5 and 168 ± 18 FNU after 15 min. Finally, in sedimentation tests with EPS from *Nitzschia* sp. cultivated using glucose as a carbon source, the initial turbidity corresponds to 903.5 ± 20.5 FNU, which decreases to 149.5 ± 5.5 and 22.2 ± 0.1 FNU for EPS–glucose of 0.1 and 1 gL^−1^, respectively (Figure 8C). When determining flocculation efficiency at 15 min, the highest efficiency is observed for EPS of 1 gL^−1^ from *Nitzschia* sp. cultivated in glucose, reaching 98.89 ± 0.1% and surpassing the 89.23 ± 1.59% efficiency obtained with SNF604 0.1 gL^−1^ (Figure 8D).

**Figure 9:**
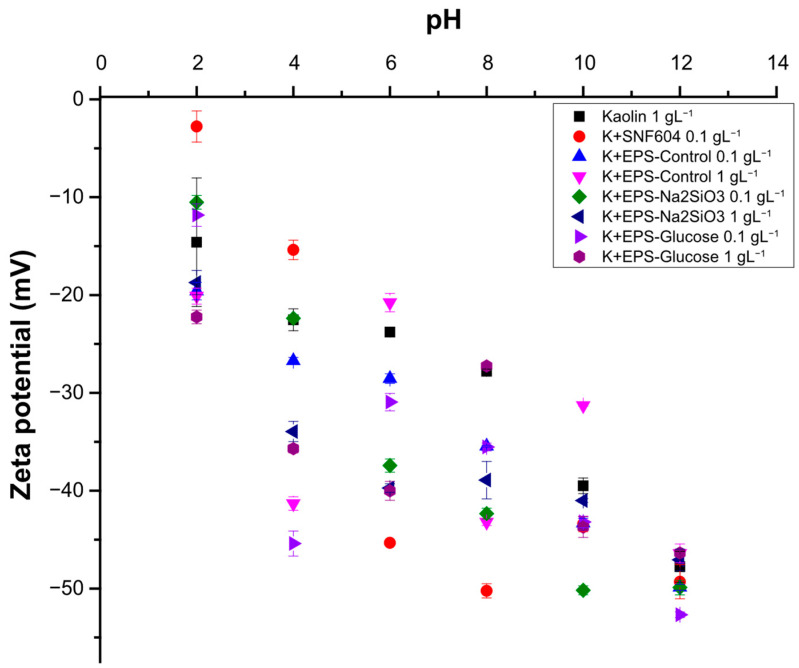
Zeta potential in deionized water of kaolinite interactions with EPS in different treatments. Kaolinite, kaolinite + SNF604, kaolinite + EPS control, kaolinite + EPS sodium metasilicate, kaolinite + EPS Glucose. K+ corresponding to Kaolinite-added EPS. Bars indicate standard error of triplicate experiments.

Subsequently, the zeta potential of flocs formed by the interaction of kaolinite with EPS from the different treatments was measured to evaluate colloidal stability. Measurements were performed in deionized water over a pH range of 2.0–12. As evidenced in Figure 9, kaolinite alone exhibited a zeta potential of approximately −15 mV at pH 2.0, decreasing to about −50 mV at pH 8.0. However, interactions of kaolinite with EPS from *Nitzschia* sp. cultivated under different conditions produced distinct zeta-potential profiles, indicating differences in surface functional groups. The interaction of the chemical flocculant SNF604 with kaolinite was also investigated, and variations in zeta potential ranging from −3 to −50 mV across pH 2.0–12. Eventually, from pH 4.0 onward, the system entered a region associated with colloidal stability, which limits particle aggregation, although the high molecular weight of commercial polymers still promotes clay flocculation and sedimentation.

Flocs formed between kaolinite and EPS from *Nitzschia* sp. cultivated under control conditions exhibited zeta potentials between −20 and −50 mV across pH 2.0–12, with abrupt variations that rendered the particles either unstable or stable depending on pH. Consequently, at pH 2.0 and pH 6.0, these interactions are prone to instability and may favor aggregation. Then, flocs formed by kaolinite and EPS from sodium metasilicate-treated cultures were investigated, resulting in zeta potentials from −10 to −50 mV over pH 2.0–12, following a predictable pH-dependent trend and remaining more negative than −30 mV from pH 6.0 to 12. Within the pH range 6.0–8.0, the interaction is constrained within the instability zone, thereby limiting aggregation and flocculation of colloidal particles more than in other treatments.

Finally, flocs formed by kaolinite and EPS from glucose-treated cultures yielded zeta potential between −11 and −53 mV across pH 2.0–12. pH 2.0 and 8.0 were identified as the most favorable forms promoting particle aggregation, corroborating the high flocculation efficiency observed with EPS of 1 gL^−1^ from *Nitzschia* cultivated with glucose of 0.1 gL^−1^. In addition, it is also possible to show that the zeta potential of kaolinite decreases at all pH levels evaluated when combined with EPS–glucose, except at pH 8.0, where the values are similar (Kaolinite corresponding to −27.8 mV and Kaolinite + EPS–glucose of 1 gL^−1^ corresponding to −27.3 mV).

**Figure 10:**
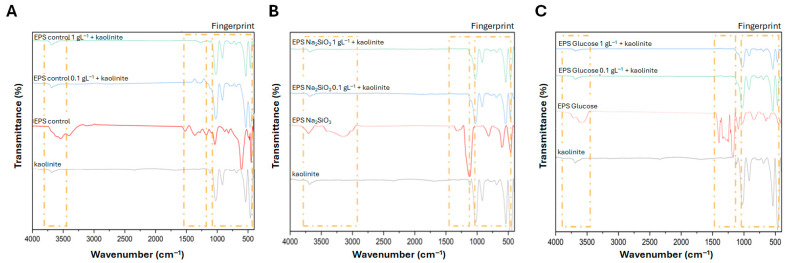
FT-IR spectra of flocs with kaolinite interactions with (**A**) EPS from *Nitzschia* sp. cultivated in control conditions; (**B**) EPS from *Nitzschia* sp. cultivated in 0.03 gL^−1^ sodium metasilicate; (**C**) EPS from *Nitzschia* sp. cultivated in 0.1 gL^−1^ glucose.

FT-IR analyses were performed on the flocs formed in the various sedimentation tests, including the positive control of clay mineral kaolinite, the chemical flocculant SNF604, and the different EPS samples (Figure 10). The spectra reveal regions associated with functional groups from both kaolinite and the EPS, indicating sites capable of mutual interaction, in addition to exhibiting fingerprints in all interactions.

FT-IR was also used to assess whether cultivation of *Nitzschia* sp. cultivated with different nutrients at different concentrations altered EPS functional groups. The spectra display prominent peaks between the ranges 3700–2800 cm^−1^ and 1600–1000 cm^−1^ (Figure 10). The absorption bands of EPS from the different treatments (control, sodium metasilicate, and glucose) indicate hydroxyl (−OH) groups at 3700–3550 cm^−1^. All samples show IR absorbance near 1100 cm^−1^, consistent with carboxylic ester (C=O) or carboxylate anion (COO−) functionalities, an important marker used to characterize different polysaccharides [53], and which may also relate to C–O and C–O–C stretching. Bands detected between 810 and 460 cm^−1^ are characteristic of the presence of glycosidic bonds [54]. Furthermore, the band near 3130 cm^−1^ in EPS from *Nitzschia* sp. culture with sodium metasilicate (0.03 gL^−1^) corresponds to the antisymmetric CH stretching vibration (Figure 10B). A distinctive pattern of peaks in 1400–1170 cm^−1^ was observed for EPS from glucose-cultured *Nitzschia* sp. (Figure 10C), attributable to CH_3_ bending (1455–1470 cm^−1^), COO− stretching (1400–1420 cm^−1^), S=O (1245–1255 cm^−1^), and O−Acetyl (1240–1250 cm^−1^) vibrations, in agreement with Borjas-Esqueda et al. [55].

Also, as shown in Figure 10A, EPS from *Nitzschia* sp. under control conditions exhibited hydroxyl (−OH) functional groups at 3700–3550 cm^−1^, and these groups interacted with kaolinite, producing attenuation of the corresponding spectral band. A similar effect was observed near 1100 cm^−1^, consistent with carboxylic ester (C=O) or carboxylate anion (COO−) functionalities in the EPS. Upon interaction with kaolinite, these bands were either attenuated or manifested as altered peak shapes. In Figure 10B, EPS from cultures treated with sodium metasilicate (0.03 gL^−1^) exhibited hydroxyl (−OH) bands at 3700–3550 cm^−1^ and C–H related bands near 3130 cm^−1^. In a similar manner, these functional groups likewise interact with kaolinite functional groups, producing comparable spectral changes. Likewise, EPS from glucose-treated cultures (Figure 10C) showed analogous interactions with kaolinite. Unlike the other EPS samples, however, the glucose-derived EPS present distinct peaks in the 1500–900 cm^−1^ region; these peaks were significantly attenuated in the flocs formed with kaolinite. The attenuated bands are attributable to O–acetyl groups near 1240 cm^−1^, uronic acids at 1446 cm^−1^, and a peak characteristic of guluronic acids at 961 cm^−1^ (Figure 10C).

## 4. Discussion

In this study, *Nitzschia* sp. strain 53.3 has been successfully isolated and described morphologically, and its stationary-phase biomass contains lipids, carbohydrates, proteins, phenolic compounds and pigments, including fucoxanthin. The EPS produced by the strain comprised polysaccharides (rhamnose, fucose, arabinose, xylose and glucose) without detectable protein or nucleic acid, and surface examination by electronic microscopy (SEM) showed a three-dimensional structure (hexagonal patterns and amorphous interlayers) that probably provided a larger surface area for interaction of those molecular structures.

A multivariate approach based on a Box–Behnken design was initially applied to evaluate the combined effects of pH, sodium carbonate, and sodium metasilicate on biomass and EPS production. While the model showed a good fit for biomass, it did not provide a statistically robust or predictive model for EPS production. Interaction effects were formally evaluated but did not show consistent or interpretable contributions within the tested experimental range. This behavior suggests that EPS biosynthesis in *Nitzschia* sp. may be governed by nonlinear responses or condition-specific effects not captured within the selected design space. Under these conditions, the multivariate framework offered limited predictive value for EPS optimization. Consequently, a targeted one-factor-at-a-time (OFAT) approach was subsequently employed as a screening strategy to identify nutrient conditions that enhance EPS productivity, a sequential strategy that has also been reported in microalgae systems [46]. Supplementation of cultures with sodium metasilicate, sodium carbonate or glucose significantly influenced growth rates and profit for EPS (concentration- and phase-dependent manner); highest growth rates and EPS productivity occurred at specific nutrient concentrations, whereas EPS yields were generally greater in the exponential phase (day 7) and declined by the stationary phase (day 17). Only certain treatments (specifically 0.03 gL^−1^ sodium metasilicate and 0.1 gL^−1^ glucose) maintained EPS production of high quality. Surface analyses indicated that EPS zeta potentials varied from −5 to −53 mV at pH 2.0–12, indicating a general tendency toward aggregation with treatment-dependent profiles; EPS from *Nitzschia* sp. cultivated with glucose presents a zeta potential (~−30 to −40 mV at pH 6.0–8.0) consistent with increased flocculation.

The sedimentation assays conducted in this study revealed that EPS synthesized by *Nitzschia* sp. strain 53.3 are capable of rapidly destabilizing kaolinite suspensions. Initially, kaolinite dispersions presented turbidity values exceeding ~2000 FNU; however, once EPS were introduced, turbidity decreased sharply within the first minutes of the assay. In particular, EPS obtained from cultures supplemented with glucose reduced turbidity to values close to 20–30 FNU after approximately fifteen minutes, corresponding to flocculation efficiencies approaching 99%. These results are notable considering that kaolinite particles normally remain suspended in aqueous environments due to their small particle size and persistent negative surface charge, characteristics that generate highly stable colloidal systems in mineral processing waters [4,7].

Similar results have been reported for microbial polymers as bioflocculants, though efficiencies usually fall within narrower ranges. EPS from microalgae reach 80–87% for biomass harvesting [56]. Bacterial EPS for clay removal or wastewater clarification range from 70 to 95%, depending on polymer composition and ionic conditions [13,15]. Within this context, the EPS obtained from *Nitzschia* sp. strain 53.3 appears to operate within the upper range of biological flocculants reported so far. Such performance becomes particularly relevant when compared with conventional synthetic polymers such as polyacrylamides, which, although highly efficient, may release residual monomers or increase the ionic strength of treated waters [16,17].

An interesting feature emerging from the present results is that the most efficient EPS were not produced under the culture conditions that maximized biomass growth. Cultures supplemented with low glucose concentrations (0.1 gL^−1^) produced EPS with the highest flocculation capacity despite exhibiting only moderate biomass productivity. This observation suggests that the effectiveness of the polymer is linked less to biomass yield and more to structural characteristics of the EPS themselves. Microalgae frequently modify the composition and architecture of extracellular polysaccharides in response to environmental factors such as nutrient availability, carbon supply or metabolic stress [36]. In diatoms, these metabolic adjustments are supported by diverse carbohydrate synthesis pathways that enable the production of structurally heterogeneous polymers.

The genomic analysis performed for *Nitzschia* sp. strain 53.3 provides indirect evidence supporting this interpretation. A large number of genes related to carbohydrate metabolism were identified, including numerous glycosyltransferases, glycoside hydrolases and enzymes such as UDP-glucose-4-epimerase, all of which participated in the synthesis and modification of polysaccharides. These enzymes belong to the group of carbohydrate-active enzymes (CAZymes), responsible for assembling monosaccharides into complex polymeric structures. Similar enzymatic repertoires have been described in other diatoms, where they contribute to the synthesis of extracellular polysaccharides involved in sediment stabilization and biofilm formation [57,58]. Consequently, minor variations in carbon availability—such as the addition of glucose at low concentrations—may redirect metabolic flux toward specific glycosylation pathways, producing polymers with distinct functional properties.

The chemical characteristics of the EPS identified in this study provide additional support for this interpretation. FTIR spectra revealed the presence of functional groups commonly associated with polysaccharides, including hydroxyl groups, carboxylates, uronic acids and O–acetyl groups. These functional groups are known to participate in different types of interactions with mineral surfaces. Carboxyl groups, for example, may act as ligands capable of binding metal ions or interacting with positively charged sites on clay particles, while hydroxyl groups can establish hydrogen bonds with mineral surfaces [31,58]. In natural environments, interactions of this kind allow microbial EPS to contribute to sediment stabilization by binding mineral grains into cohesive aggregates [27,34].

A particularly interesting feature observed in the present study was the behavior of EPS obtained from glucose-supplemented cultures. The FT-IR spectra of these polymers showed distinctive signals in the region between roughly 1400 and 900 cm^−1^, commonly associated with uronic acids, carboxylate groups and acetylated polysaccharides. After contact with kaolinite, several of these bands appeared attenuated or slightly shifted, suggesting that the corresponding functional groups participated directly in the aggregation process. Comparable spectral variations have been described in systems where microbial polysaccharides interact with clay minerals through ligand exchange or hydrogen bonding mechanisms [58].

The electrostatic properties of the EPS–kaolinite system also provide insight into the aggregation mechanism. Zeta potential measurements showed that EPS and EPS–clay aggregates remained within a moderately unstable range, generally between approximately −10 and −30 mV across the evaluated pH ranges. Suspensions with absolute zeta potential values above ±30 mV are typically considered electrostatically stable; therefore, the intermediate values observed here suggest that aggregation cannot be explained solely by charge neutralization. Instead, the data point toward a mechanism dominated by polymer bridging, in which long polysaccharide chains adsorb simultaneously onto multiple particles, creating interparticle connections that promote floc formation. Bridging mechanisms of this type are widely described for high-molecular-weight polymers used as flocculants in mineral processing systems [14,15].

The structural characteristics of the EPS further support this interpretation. SEM observations revealed a three-dimensional architecture composed of hexagonal patterns and branched polymer structures with dimensions on the order of several micrometers. Such morphology increases the surface area available for particle interactions and facilitates entanglement between clay particles. Branched EPS structures have been described previously in several microalgae and microorganisms, where they form complex networks capable of trapping suspended particles and enhancing aggregation processes [59]. In cyanobacteria such as *Mycrocystis aeruginosa*, for example, extracellular polymers form porous networks that physically retain particles within the polymer matrix [29].

These structural characteristics may also help explain the rapid kinetics observed during sedimentation experiments. In several treatments, turbidity decreased markedly during the first five minutes of the assay, suggesting that once EPS encounters suspended particles, adsorption and aggregation occur quickly. Rapid floc formation is advantageous in industrial clarification processes because it reduces settling times and improves the efficiency of thickening operations.

From a practical perspective, these results highlight the potential of diatom-derived EPS as bioflocculants for treating recirculated mining waters. Copper mining operations increasingly rely on water recirculation systems, particularly in arid regions such as northern Chile, where freshwater resources are limited [1,2]. However, the accumulation of fine clay particles in these circuits can interfere with flotation processes and reduce mineral recovery [4]. The use of biodegradable polymers such as EPS may therefore represent a sustainable alternative to synthetic flocculants in these systems.

Nevertheless, several aspects require further investigation before large-scale application can be considered. Mining waters often contain elevated concentrations of dissolved salts and metal ions that may alter polymer conformation or interfere with clay–polymer interactions. In some cases, metal ions may enhance flocculation through cation bridging; in others, they may inhibit polymer adsorption depending on their concentration and speciation. For example, the presence of cations such as Na^+^ and K^+^ will alter the surface properties of clays such as kaolinite. The ion acts as a “breaker ion” that affects the zeta potential of the clay, while promoting the formation of a hydrated layer that favors the stability of the particles in suspension, hindering their sedimentation [60]. On the other hand, calcium is essential for aiding the dewatering process of tailings rich in expansive clays [61]. Other ions, such as chloride, are critical when using seawater or brackish wáter. Chloride affects the dissolution of electrodes (such as titanium) in electroflotation processes, causing pitting corrosion and facilitating the formation of electrochemical coagulants. It also influences the stability of clay suspensions [60]. In addition, the economic feasibility of producing EPS at industrial scale will depend on optimizing culture conditions, extraction methods and polymer recovery.

The results obtained in this study suggest that metabolic modulation through low-level glucose supplementation induces subtle structural changes in the EPS produced by *Nitzschia* sp. strain 53.3. These modifications appear to increase the density of functional groups capable of interacting with kaolinite surfaces, ultimately enhancing polymer–particle affinity and promoting efficient flocculation.

## 5. Conclusions

In kaolinite sedimentation tests, the EPS doses of 0.9 and 1.0 gL^−1^ led to rapid turbidity reductions and the highest flocculation efficiencies at 15 min, reaching up to ~96.7% flocculation efficiency for EPS from glucose-cultured cells, achieving or exceeding the final flocculation efficiency of the commercial flocculant SNF604, although the chemical flocculant had a higher flocculation rate at early time points.

FT-IR characterization of EPS–kaolinite flocs showed interacting functional regions, the attenuation of hydroxyl bands (3700–3550 cm^−1^), C=O/COO– and C–O stretches (~1100 cm^−1^), and Si–O–Si and Si–O–Al signatures of kaolinite (1120–910, 680, 530, 460 cm^−1^), and showed that glucose-derived EPS present unique O–acetyl, uronic and guluronic signals (1500–900 cm^−1^) that were significantly attenuated by floc formation, indicating that they removed more turbid particles.

Overall, the data indicate that EPS composition and structure are very sensitive to culture conditions and nutrient supply, and that these differences significantly modulate interactions with clay particles, colloidal stability and flocculation performance; EPS produced with glucose appeared especially promising in the context of kaolinite sedimentation as bioflocculants.

## Figures and Tables

**Table 1:** Nutrient concentrations used for the cultivation of *Nitzschia* sp. strain 53.3.

Variable	Level 1 gL^−1^	Level 2 gL^−1^	Level 3 gL^−1^	Level 4 gL^−1^	Level 5 gL^−1^	Level 6 gL^−1^
Sodium carbonate	0.03	0.3	1.0	1.5	2.0	2.5
Glucose	0.1	1.0	1.5	2.0	2.5	-
Sodium metasilicate	0.02	0.2	1.0	1.5	2.0	2.5

## Data Availability

All data generated in this study will be made available upon request.

## Supplementary Materials

The following supporting information can be downloaded at: https://www.mdpi.com/article/10.3390/polym18101221/s1, Figure S1: Response surface plots, biomass analysis; Figure S2: Response surface plots, EPS dry weight analysis; Table S1: Frequency of genes involved in carbon utilization; Table S2: Factors and levels for DBB; Table S3: Resulting combinations correspond to the different growing conditions; Table S4: EPS productivity (mg·L^−1^·d^−1^) of *Nitzschia* sp. strain 53.3 under different sodium metasilicate, glucose, and sodium carbonate treatments. Data corresponds to measurements taken on days 7 and 17.
