# Supplementary material for: Extracellular Polymers from Nitzschia sp. for Removing Clay Minerals from Water in Mining
Source: Polymers (Basel). 2026 May 17;18(10):1221. doi: 10.3390/polym18101221 (PMC13210723; doi:10.3390/polym18101221)
Supplement: Supplementary file 1 [file polymers-18-01221-s001.zip › Table S1 revMR.pdf]

Table S1. Frequency of genes involved in carbon utilization.

gene_id	header	subheader	module	gene_description
**GH5**	CAZY	mixed-Linkage glucans Backbone Cleavage (Hemicellulose), Chitin Oligo Cleavage	Glycoside Hydrolases	beta-D-galactofuranosidase (EC 3.2.1.146);
**GH16**	CAZY	Sulf-Polysachharides Backbone Cleavage	Glycoside Hydrolases	endo-beta-1,3-galactanase (EC 3.2.1.181)
**PL1**	CAZY	Pectin Backbone Cleavage	Polysaccharide Lyases	PL1 pectate lyase (EC 4.2.2.2); exo-pectate lyase (EC 4.2.2.9); pectin lyase (EC 4.2.2.10).
**PL10**	CAZY	Pectin Backbone Cleavage	Polysaccharide Lyases	PL10 pectate lyase (EC 4.2.2.2)
**PL11**	CAZY	Pectin Backbone Cleavage	Polysaccharide Lyases	PL11 rhamnogalacturonan endolyase (EC 4.2.2.23); rhamnogalacturonan exolyase (EC 4.2.2.24)
**PL9**	CAZY	Pectin Backbone Cleavage	Polysaccharide Lyases	PL9 pectate lyase (EC 4.2.2.2); exopolygalacturonate lyase (EC 4.2.2.9); thiopeptidoglycan lyase (EC 4.2.2.-).
**GH13**	CAZY	Starch Backbone Cleavage	Glycoside Hydrolases	GH13 alpha-amylase (EC 3.2.1.1)
**PL12**	CAZY	Sulf-Polysachharides Backbone Cleavage	Polysaccharide Lyases	PL12 heparin-sulfate lyase (EC 4.2.2.8)
**PL33**	CAZY	Sulf-Polysachharides Backbone Cleavage	Polysaccharide Lyases	PL33 hyaluronate lyase (EC 4.2.2.1); gellan lyase (EC 4.2.2.25); chondroitin sulfate lyase (EC 4.2.2.20)
**PL8**	CAZY	Sulf-Polysachharides Backbone Cleavage	Polysaccharide Lyases	PL8 hyaluronate lyase (EC 4.2.2.1); chondroitin AC lyase (EC 4.2.2.5); xanthan lyase (EC 4.2.2.12); chondroitin ABC lyase (EC 4.2.2.20)
**PL6**	CAZY	Sulf-Polysachharides Backbone Cleavage, Sulf-Polysachharides Oligo Cleavage	Polysaccharide Lyases	PL6 alginate lyase (EC 4.2.2.3); chondroitinase B (EC 4.2.2.19); MG-specific alginate lyase (EC 4.2.2.-); poly(alpha-L-guluronate) lyase / G-specific alginate lyase (EC 4.2.2.11);
**GH30**	CAZY	Xylan Backbone Cleavage (Hemicellulose)	Glycoside Hydrolases	GH30 endo-beta-1,4-xylanase (EC 3.2.1.8)
**GH43**	CAZY	Xyloglucan Oligo Cleavage (Hemicellulose), Arabinan Backbone Cleavage, Arabinan Oligo Cleavage	Glycoside Hydrolases	GH43 beta-xylosidase (EC 3.2.1.37); alpha-L-arabinofuranosidase (EC 3.2.1.55); xylanase (EC 3.2.1.8); alpha-1,2-L-arabinofuranosidase (EC 3.2.1.-); exo-alpha-1,5-L-arabinofuranosidase (EC 3.2.1.-); [inverting] exo-alpha-1,5-L-arabinanase (EC 3.2.1.-); beta-1,3-xylosidase (EC 3.2.1.-); [inverting] exo-alpha-1,5-L-arabinanase (EC 3.2.1.-); [inverting] endo-alpha-1,5-L-arabinanase (EC 3.2.1.99); exo-beta-1,3-galactanase (EC 3.2.1.145); beta-D-galactofuranosidase (EC 3.2.1.146)
**AA3**	CAZY		Auxiliary Activities	AA3 cellobiose dehydrogenase (EC 1.1.99.18); glucose 1-oxidase (EC 1.1.3.4); aryl alcohol oxidase (EC 1.1.3.7); alcohol oxidase (EC 1.1.3.13); pyranose oxidase (EC 1.1.3.10)

Table S1. Frequency of genes involved in carbon utilization.

**AA5**	CAZY		Auxiliary Activities	AA5 Oxidase with oxygen as acceptor (EC 1.1.3.-); galactose oxidase (EC 1.1.3.9); glyoxal oxidase (EC 1.2.3.15); alcohol oxidase (EC 1.1.3.13)
**GT2**	CAZY		GlycosylTransferases	GT2 cellulose synthase (EC 2.4.1.12); chitin synthase (EC 2.4.1.16); dolichyl-phosphate beta-D-mannosyltransferase (EC 2.4.1.83); dolichyl-phosphate beta-glucosyltransferase (EC 2.4.1.117); N-acetylglucosaminyltransferase (EC 2.4.1.-); N-acetylgalactosaminyltransferase (EC 2.4.1.-); hyaluronan synthase (EC 2.4.1.212); chitin oligosaccharide synthase (EC 2.4.1.-); beta-1,3-glucan synthase (EC 2.4.1.34); beta-1,4-mannan synthase (EC 2.4.1.-); beta-mannosylphosphodecaprenol-mannooligosaccharide alpha-1,6-mannosyltransferase (EC 2.4.1.199); UDP-Galf: rhamnopyranosyl-N-acetylglucosaminyl-PP-decaprenol beta-1,4/1,5-galactofuranosyltransferase (EC 2.4.1.287); UDP-Galf: galactofuranosyl-galactofuranosyl-rhamnosyl-N-acetylglucosaminyl-PP-decaprenol beta-1,5/1,6-galactofuranosyltransferase (EC 2.4.1.288); dTDP-L-Rha: N-acetylglucosaminyl-PP-decaprenol alpha-1,3-L-rhamnosyltransferase (EC 2.4.1.289)
**PL14**	CAZY		Polysaccharide Lyases	PL14 alginate lyase (EC 4.2.2.3); exo-oligoalginate lyase (EC 4.2.2.26); beta-1,4-glucuronan lyase (EC 4.2.2.14)
**PL17**	CAZY		Polysaccharide Lyases	PL17 alginate lyase (EC 4.2.2.3); oligoalginate lyase (EC 4.2.2.26)
**PL7**	CAZY		Polysaccharide Lyases	PL7 poly(beta-mannuronate) lyase / M-specific alginate lyase (EC 4.2.2.3); alpha-L-guluronate lyase / G-specific alginate lyase (EC 4.2.2.11); poly-(MG)-lyase / MG-specific alginate lyase (EC 4.2.2.-); endo-beta-1,4-glucuronan lyase (EC 4.2.2.14)
**K00134**	central carbon	glycolysis	D-galactonate degradation, De Ley-Doudoroff pathway, D-galactonate => glycerate-3P	glyceraldehyde 3-phosphate dehydrogenase [EC:1.2.1.12] [RN:R01061]
**K00883**	central carbon	glycolysis	D-galactonate degradation, De Ley-Doudoroff pathway, D-galactonate => glycerate-3P	2-dehydro-3-deoxygalactonokinase [EC:2.7.1.58] [RN:R03387]

Table S1. Frequency of genes involved in carbon utilization.

**K00927**	central carbon	glycolysis	D-galactonate degradation, De Ley-Doudoroff pathway, D-galactonate => glycerate-3P	phosphoglycerate kinase [EC:2.7.2.3] [RN:R01512]
**K01631**	central carbon	glycolysis	D-galactonate degradation, De Ley-Doudoroff pathway, D-galactonate => glycerate-3P	2-dehydro-3-deoxyphosphogalactonate aldolase [EC:4.1.2.21] [RN:R01064]
**K01684**	central carbon	glycolysis	D-galactonate degradation, De Ley-Doudoroff pathway, D-galactonate => glycerate-3P	galactonate dehydratase [EC:4.2.1.6] [RN:R03033]
**K00041**	central carbon	glycolysis	D-Galacturonate degradation (bacteria), D-galacturonate => pyruvate + D-glyceraldehyde 3P	tagaturonate reductase [EC:1.1.1.58] [RN:R02555]
**K00874**	central carbon	glycolysis	D-Galacturonate degradation (bacteria), D-galacturonate => pyruvate + D-glyceraldehyde 3P	2-dehydro-3-deoxygluconokinase [EC:2.7.1.45] [RN:R01541]
**K01625**	central carbon	glycolysis	D-Galacturonate degradation (bacteria), D-galacturonate => pyruvate + D-glyceraldehyde 3P	2-dehydro-3-deoxy-phosphogluconate aldolase [EC:4.1.2.14] [RN:R05605]
**K01685**	central carbon	glycolysis	D-Galacturonate degradation (bacteria), D-galacturonate => pyruvate + D-glyceraldehyde 3P	altronate hydrolase [EC:4.2.1.7] [RN:R01540]

Table S1. Frequency of genes involved in carbon utilization.

**K01812**	central carbon	glycolysis	D-Galacturonate degradation (bacteria), D-galacturonate => pyruvate + D-glyceraldehyde 3P	glucuronate isomerase [EC:5.3.1.12] [RN:R01983]
**K00036**	central carbon	glycolysis	Entner-Doudoroff pathway, glucose-6P => glyceraldehyde-3P + pyruvate	glucose-6-phosphate 1-dehydrogenase [EC:1.1.1.49 1.1.1.363] [RN:R02736 R10907]
**K01057**	central carbon	glycolysis	Entner-Doudoroff pathway, glucose-6P => glyceraldehyde-3P + pyruvate	6-phosphogluconolactonase [EC:3.1.1.31] [RN:R02035]
**K01625**	central carbon	glycolysis	Entner-Doudoroff pathway, glucose-6P => glyceraldehyde-3P + pyruvate	2-dehydro-3-deoxyphosphogluconate aldolase [EC:4.1.2.14] [RN:R05605]
**K01690**	central carbon	glycolysis	Entner-Doudoroff pathway, glucose-6P => glyceraldehyde-3P + pyruvate	phosphogluconate dehydratase [EC:4.2.1.12] [RN:R02036]
**K07404**	central carbon	glycolysis	Entner-Doudoroff pathway, glucose-6P => glyceraldehyde-3P + pyruvate	6-phosphogluconolactonase [EC:3.1.1.31] [RN:R02035]
**K00849**	central carbon	glycolysis	Galactose degradation, Leloir pathway, galactose => alpha-D-glucose-1P	galactokinase [EC:2.7.1.6] [RN:R01092]
**K01784**	central carbon	glycolysis	Galactose degradation, Leloir pathway, galactose => alpha-D-glucose-1P	UDP-glucose 4-epimerase [EC:5.1.3.2] [RN:R00291]
**K01785**	central carbon	glycolysis	Galactose degradation, Leloir pathway,	aldose 1-epimerase [EC:5.1.3.3] [RN:R10619]

Table S1. Frequency of genes involved in carbon utilization.

			galactose => alpha-D-glucose-1P	
**K00134**	central carbon	glycolysis	Gluconeogenesis, oxaloacetate => fructose-6P	glyceraldehyde 3-phosphate dehydrogenase [EC:1.2.1.12 1.2.1.59] [RN:R01061 R01063]
**K00150**	central carbon	glycolysis	Gluconeogenesis, oxaloacetate => fructose-6P	glyceraldehyde 3-phosphate dehydrogenase [EC:1.2.1.12 1.2.1.59] [RN:R01061 R01063]
**K00927**	central carbon	glycolysis	Gluconeogenesis, oxaloacetate => fructose-6P	phosphoglycerate kinase [EC:2.7.2.3] [RN:R01512]
**K01596**	central carbon	glycolysis	Gluconeogenesis, oxaloacetate => fructose-6P	phosphoenolpyruvate carboxykinase [EC:4.1.1.32 4.1.1.49] [RN:R00431 R00726 R00341]
**K01610**	central carbon	glycolysis	Gluconeogenesis, oxaloacetate => fructose-6P	phosphoenolpyruvate carboxykinase [EC:4.1.1.32 4.1.1.49] [RN:R00431 R00726 R00341]
**K01623**	central carbon	glycolysis	Gluconeogenesis, oxaloacetate => fructose-6P	fructose-bisphosphate aldolase [EC:4.1.2.13] [RN:R01070]
**K01624**	central carbon	glycolysis	Gluconeogenesis, oxaloacetate => fructose-6P	fructose-bisphosphate aldolase [EC:4.1.2.13] [RN:R01070]
**K01689**	central carbon	glycolysis	Gluconeogenesis, oxaloacetate => fructose-6P	enolase [EC:4.2.1.11] [RN:R00658]
**K01803**	central carbon	glycolysis	Gluconeogenesis, oxaloacetate => fructose-6P	triosephosphate isomerase (TIM) [EC:5.3.1.1] [RN:R01015]
**K01834**	central carbon	glycolysis	Gluconeogenesis, oxaloacetate => fructose-6P	phosphoglycerate mutase [EC:5.4.2.11 5.4.2.12] [RN:R01518]
**K03841**	central carbon	glycolysis	Gluconeogenesis, oxaloacetate => fructose-6P	fructose-1,6-bisphosphatase [EC:3.1.3.11] [RN:R04780]

Table S1. Frequency of genes involved in carbon utilization.

**K04041**	central carbon	glycolysis	Gluconeogenesis, oxaloacetate => fructose-6P	fructose-1,6-bisphosphatase [EC:3.1.3.11] [RN:R04780]
**K11532**	central carbon	glycolysis	Gluconeogenesis, oxaloacetate => fructose-6P	fructose-1,6-bisphosphatase [EC:3.1.3.11] [RN:R04780]
**K11645**	central carbon	glycolysis	Gluconeogenesis, oxaloacetate => fructose-6P	fructose-bisphosphate aldolase [EC:4.1.2.13] [RN:R01070]
**K15633**	central carbon	glycolysis	Gluconeogenesis, oxaloacetate => fructose-6P	phosphoglycerate mutase [EC:5.4.2.11 5.4.2.12] [RN:R01518]
**K15635**	central carbon	glycolysis	Gluconeogenesis, oxaloacetate => fructose-6P	phosphoglycerate mutase [EC:5.4.2.11 5.4.2.12] [RN:R01518]
**K00134**	central carbon	glycolysis	Glycolysis (Embden-Meyerhof pathway), glucose => pyruvate	glyceraldehyde 3-phosphate dehydrogenase [EC:1.2.1.12 1.2.1.59] [RN:R01061 R01063]
**K00150**	central carbon	glycolysis	Glycolysis (Embden-Meyerhof pathway), glucose => pyruvate	glyceraldehyde 3-phosphate dehydrogenase [EC:1.2.1.12 1.2.1.59] [RN:R01061 R01063]
**K00845**	central carbon	glycolysis	Glycolysis (Embden-Meyerhof pathway), glucose => pyruvate	hexokinase/glucokinase [EC:2.7.1.1 2.7.1.2] [RN:R01786]
**K00850**	central carbon	glycolysis	Glycolysis (Embden-Meyerhof pathway), glucose => pyruvate	6-phosphofructokinase [EC:2.7.1.11] [RN:R04779]
**K00873**	central carbon	glycolysis	Glycolysis (Embden-Meyerhof pathway), glucose => pyruvate	pyruvate kinase [EC:2.7.1.40] [RN:R00200]
**K00886**	central carbon	glycolysis	Glycolysis (Embden-Meyerhof pathway), glucose => pyruvate	polyphosphate glucokinase [EC:2.7.1.63] [RN:R02189]
**K00927**	central carbon	glycolysis	Glycolysis (Embden-Meyerhof pathway), glucose => pyruvate	phosphoglycerate kinase [EC:2.7.2.3] [RN:R01512]

Table S1. Frequency of genes involved in carbon utilization.

**K01623**	central carbon	glycolysis	Glycolysis (Embden-Meyerhof pathway), glucose => pyruvate	fructose-bisphosphate aldolase [EC:4.1.2.13] [RN:R01070]
**K01624**	central carbon	glycolysis	Glycolysis (Embden-Meyerhof pathway), glucose => pyruvate	fructose-bisphosphate aldolase [EC:4.1.2.13] [RN:R01070]
**K01689**	central carbon	glycolysis	Glycolysis (Embden-Meyerhof pathway), glucose => pyruvate	enolase [EC:4.2.1.11] [RN:R00658]
**K01803**	central carbon	glycolysis	Glycolysis (Embden-Meyerhof pathway), glucose => pyruvate	triosephosphate isomerase [EC:5.3.1.1] [RN:R01015]
**K01810**	central carbon	glycolysis	Glycolysis (Embden-Meyerhof pathway), glucose => pyruvate	glucose-6-phosphate isomerase [EC:5.3.1.9] [RN:R02740]
**K01834**	central carbon	glycolysis	Glycolysis (Embden-Meyerhof pathway), glucose => pyruvate	phosphoglycerate mutase [EC:5.4.2.11 5.4.2.12] [RN:R01518]
**K06859**	central carbon	glycolysis	Glycolysis (Embden-Meyerhof pathway), glucose => pyruvate	glucose-6-phosphate isomerase [EC:5.3.1.9] [RN:R02740]
**K11645**	central carbon	glycolysis	Glycolysis (Embden-Meyerhof pathway), glucose => pyruvate	fructose-bisphosphate aldolase [EC:4.1.2.13] [RN:R01070]
**K15633**	central carbon	glycolysis	Glycolysis (Embden-Meyerhof pathway), glucose => pyruvate	phosphoglycerate mutase [EC:5.4.2.11 5.4.2.12] [RN:R01518]
**K15635**	central carbon	glycolysis	Glycolysis (Embden-Meyerhof pathway), glucose => pyruvate	phosphoglycerate mutase [EC:5.4.2.11 5.4.2.12] [RN:R01518]
**K15916**	central carbon	glycolysis	Glycolysis (Embden-Meyerhof pathway), glucose => pyruvate	glucose-6-phosphate isomerase [EC:5.3.1.9] [RN:R02740]
**K16370**	central carbon	glycolysis	Glycolysis (Embden-Meyerhof pathway), glucose => pyruvate	6-phosphofructokinase [EC:2.7.1.11] [RN:R04779]

Table S1. Frequency of genes involved in carbon utilization.

**K00134**	central carbon	glycolysis	Glycolysis, core module involving three-carbon compounds	glyceraldehyde 3-phosphate dehydrogenase [EC:1.2.1.12 1.2.1.59] [RN:R01061 R01063]
**K00150**	central carbon	glycolysis	Glycolysis, core module involving three-carbon compounds	glyceraldehyde 3-phosphate dehydrogenase [EC:1.2.1.12 1.2.1.59] [RN:R01061 R01063]
**K00873**	central carbon	glycolysis	Glycolysis, core module involving three-carbon compounds	pyruvate kinase [EC:2.7.1.40] [RN:R00200]
**K00927**	central carbon	glycolysis	Glycolysis, core module involving three-carbon compounds	phosphoglycerate kinase [EC:2.7.2.3] [RN:R01512]
**K01689**	central carbon	glycolysis	Glycolysis, core module involving three-carbon compounds	enolase [EC:4.2.1.11] [RN:R00658]
**K01803**	central carbon	glycolysis	Glycolysis, core module involving three-carbon compounds	triosephosphate isomerase [EC:5.3.1.1] [RN:R01015]
**K01834**	central carbon	glycolysis	Glycolysis, core module involving three-carbon compounds	phosphoglycerate mutase [EC:5.4.2.11 5.4.2.12] [RN:R01518]
**K15633**	central carbon	glycolysis	Glycolysis, core module involving three-carbon compounds	phosphoglycerate mutase [EC:5.4.2.11 5.4.2.12] [RN:R01518]
**K15635**	central carbon	glycolysis	Glycolysis, core module involving three-carbon compounds	phosphoglycerate mutase [EC:5.4.2.11 5.4.2.12] [RN:R01518]
**K00024**	central carbon	glycolysis	Glyoxylate cycle	malate dehydrogenase [EC:1.1.1.37] [RN:R00342]
**K01637**	central carbon	glycolysis	Glyoxylate cycle	isocitrate lyase [EC:4.1.3.1] [RN:R00479]
**K01638**	central carbon	glycolysis	Glyoxylate cycle	malate synthase [EC:2.3.3.9] [RN:R00472]
**K01647**	central carbon	glycolysis	Glyoxylate cycle	citrate synthase [EC:2.3.3.1] [RN:R00351]

Table S1. Frequency of genes involved in carbon utilization.

**K01681**	central carbon	glycolysis	Glyoxylate cycle	aconitate hydratase [EC:4.2.1.3] [RN:R01325 R01900]
**K01682**	central carbon	glycolysis	Glyoxylate cycle	aconitate hydratase [EC:4.2.1.3] [RN:R01325 R01900]
**K00131**	central carbon	glycolysis	Semi-phosphorylative Entner-Doudoroff pathway, gluconate => glycerate-3P	glyceraldehyde-3-phosphate dehydrogenase (NADP+) [EC:1.2.1.9] [RN:R01058]
**K00134**	central carbon	glycolysis	Semi-phosphorylative Entner-Doudoroff pathway, gluconate => glycerate-3P	glyceraldehyde 3-phosphate dehydrogenase [EC:1.2.1.12] [RN:R01061]
**K00874**	central carbon	glycolysis	Semi-phosphorylative Entner-Doudoroff pathway, gluconate => glycerate-3P	2-dehydro-3-deoxygluconokinase [EC:2.7.1.45] [RN:R01541]
**K00927**	central carbon	glycolysis	Semi-phosphorylative Entner-Doudoroff pathway, gluconate => glycerate-3P	phosphoglycerate kinase [EC:2.7.2.3] [RN:R01512]
**K01625**	central carbon	glycolysis	Semi-phosphorylative Entner-Doudoroff pathway, gluconate => glycerate-3P	2-dehydro-3-deoxyphosphogluconate aldolase [EC:4.1.2.14] [RN:R05605]
**K00131**	central carbon	glycolysis	Semi-phosphorylative Entner-Doudoroff pathway, gluconate/galactonate => glycerate-3P	glyceraldehyde-3-phosphate dehydrogenase (NADP+) [EC:1.2.1.9] [RN:R01058]
**K01053**	central carbon	pentose pathway	Pentose phosphate pathway	gluconolactonase
**K00033**	central carbon	pentose pathway	Pentose phosphate pathway (Pentose phosphate cycle)	6-phosphogluconate dehydrogenase [EC:1.1.1.44 1.1.1.343] [RN:R01528 R10221]

Table S1. Frequency of genes involved in carbon utilization.

**K00036**	central carbon	pentose pathway	Pentose phosphate pathway (Pentose phosphate cycle)	glucose-6-phosphate 1-dehydrogenase [EC:1.1.1.49 1.1.1.363 1.1.1.388] [RN:R02736 R10907]
**K00615**	central carbon	pentose pathway	Pentose phosphate pathway (Pentose phosphate cycle)	transketolase [EC:2.2.1.1] [RN:R01641]
**K00616**	central carbon	pentose pathway	Pentose phosphate pathway (Pentose phosphate cycle)	transaldolase [EC:2.2.1.2] [RN:R01827]
**K01057**	central carbon	pentose pathway	Pentose phosphate pathway (Pentose phosphate cycle)	6-phosphogluconolactonase [EC:3.1.1.31] [RN:R02035]
**K01783**	central carbon	pentose pathway	Pentose phosphate pathway (Pentose phosphate cycle)	ribulose-phosphate 3-epimerase [EC:5.1.3.1] [RN:R01529]
**K01807**	central carbon	pentose pathway	Pentose phosphate pathway (Pentose phosphate cycle)	ribose 5-phosphate isomerase [EC:5.3.1.6] [RN:R01056]
**K01808**	central carbon	pentose pathway	Pentose phosphate pathway (Pentose phosphate cycle)	ribose 5-phosphate isomerase [EC:5.3.1.6] [RN:R01056]
**K01810**	central carbon	pentose pathway	Pentose phosphate pathway (Pentose phosphate cycle)	glucose-6-phosphate isomerase [EC:5.3.1.9] [RN:R02740 R02739]
**K06859**	central carbon	pentose pathway	Pentose phosphate pathway (Pentose phosphate cycle)	glucose-6-phosphate isomerase [EC:5.3.1.9] [RN:R02740 R02739]
**K07404**	central carbon	pentose pathway	Pentose phosphate pathway (Pentose phosphate cycle)	6-phosphogluconolactonase [EC:3.1.1.31] [RN:R02035]
**K15916**	central carbon	pentose pathway	Pentose phosphate pathway (Pentose phosphate cycle)	glucose-6-phosphate isomerase [EC:5.3.1.9] [RN:R02740 R02739]
**K01807**	central carbon	pentose pathway	Pentose phosphate pathway, archaea, fructose 6P => ribose 5P	ribose 5-phosphate isomerase A [EC:5.3.1.6] [RN:R01056]

Table S1. Frequency of genes involved in carbon utilization.

**K08093**	central carbon	pentose pathway	Pentose phosphate pathway, archaea, fructose 6P => ribose 5P	3-hexulose-6-phosphate synthase [EC:4.1.2.43] [RN:R05338]
**K08094**	central carbon	pentose pathway	Pentose phosphate pathway, archaea, fructose 6P => ribose 5P	6-phospho-3-hexuloisomerase [EC:5.3.1.27] [RN:R09780]
**K00615**	central carbon	pentose pathway	Pentose phosphate pathway, non-oxidative phase, fructose 6P => ribose 5P	transketolase [EC:2.2.1.1] [RN:R01830 R01641]
**K00616**	central carbon	pentose pathway	Pentose phosphate pathway, non-oxidative phase, fructose 6P => ribose 5P	transaldolase [EC:2.2.1.2] [RN:R01827]
**K01783**	central carbon	pentose pathway	Pentose phosphate pathway, non-oxidative phase, fructose 6P => ribose 5P	ribulose-phosphate 3-epimerase [EC:5.1.3.1] [RN:R01529]
**K01807**	central carbon	pentose pathway	Pentose phosphate pathway, non-oxidative phase, fructose 6P => ribose 5P	ribose 5-phosphate isomerase [EC:5.3.1.6] [RN:R01056]
**K01808**	central carbon	pentose pathway	Pentose phosphate pathway, non-oxidative phase, fructose 6P => ribose 5P	ribose 5-phosphate isomerase [EC:5.3.1.6] [RN:R01056]
**K00033**	central carbon	pentose pathway	Pentose phosphate pathway, oxidative phase, glucose 6P => ribulose 5P	6-phosphogluconate dehydrogenase [EC:1.1.1.44 1.1.1.343] [RN:R01528 R10221]
**K00036**	central carbon	pentose pathway	Pentose phosphate pathway, oxidative phase, glucose 6P => ribulose 5P	glucose-6-phosphate 1-dehydrogenase [EC:1.1.1.49 1.1.1.363 1.1.1.388] [RN:R02736 R10907]
**K01057**	central carbon	pentose pathway	Pentose phosphate pathway, oxidative	6-phosphogluconolactonase [EC:3.1.1.31] [RN:R02035]

Table S1. Frequency of genes involved in carbon utilization.

			phase, glucose 6P => ribulose 5P	
**K07404**	central carbon	pentose pathway	Pentose phosphate pathway, oxidative phase, glucose 6P => ribulose 5P	6-phosphogluconolactonase [EC:3.1.1.31] [RN:R02035]
**K00948**	central carbon	pentose pathway	PRPP biosynthesis, ribose 5P => PRPP	ribose-phosphate pyrophosphokinase [EC:2.7.6.1] [RN:R01049]
**K00024**	central carbon	TCA	Citrate cycle (TCA cycle, Krebs cycle)	malate dehydrogenase [EC:1.1.1.37] [RN:R00342]
**K00030**	central carbon	TCA	Citrate cycle (TCA cycle, Krebs cycle)	isocitrate dehydrogenase [EC:1.1.1.42 1.1.1.41] [RN:R01899 R00268 R00267 R00709]
**K00031**	central carbon	TCA	Citrate cycle (TCA cycle, Krebs cycle)	isocitrate dehydrogenase [EC:1.1.1.42 1.1.1.41] [RN:R01899 R00268 R00267 R00709]
**K00116**	central carbon	TCA	Citrate cycle (TCA cycle, Krebs cycle)	malate dehydrogenase (quinone) [EC:1.1.5.4] [RN:R00361]
**K00164**	central carbon	TCA	Citrate cycle (TCA cycle, Krebs cycle)	2-oxoglutarate dehydrogenase complex [EC:1.2.4.2 2.3.1.61 1.8.1.4] [RN:R00621 R03316 R02570 R07618]
**K00174**	central carbon	TCA	Citrate cycle (TCA cycle, Krebs cycle)	2-oxoglutarate/2-oxoacid ferredoxin oxidoreductase subunit alpha [EC:1.2.7.3 1.2.7.11] [RN:R01197]
**K00175**	central carbon	TCA	Citrate cycle (TCA cycle, Krebs cycle)	2-oxoglutarate/2-oxoacid ferredoxin oxidoreductase subunit beta [EC:1.2.7.3 1.2.7.11] [RN:R01197]
**K00239**	central carbon	TCA	Citrate cycle (TCA cycle, Krebs cycle)	succinate dehydrogenase / fumarate reductase [EC:1.3.5.1 1.3.5.4] [RN:R02164]
**K00240**	central carbon	TCA	Citrate cycle (TCA cycle, Krebs cycle)	succinate dehydrogenase / fumarate reductase [EC:1.3.5.1 1.3.5.4] [RN:R02164]
**K00241**	central carbon	TCA	Citrate cycle (TCA cycle, Krebs cycle)	succinate dehydrogenase / fumarate reductase [EC:1.3.5.1 1.3.5.4] [RN:R02164]
**K00242**	central carbon	TCA	Citrate cycle (TCA cycle, Krebs cycle)	succinate dehydrogenase / fumarate reductase [EC:1.3.5.1 1.3.5.4] [RN:R02164]
**K00244**	central carbon	TCA	Citrate cycle (TCA cycle, Krebs cycle)	fumarate reductase [EC:1.3.5.4] [RN:R02164]
**K00382**	central carbon	TCA	Citrate cycle (TCA cycle, Krebs cycle)	2-oxoglutarate dehydrogenase complex [EC:1.2.4.2 2.3.1.61 1.8.1.4] [RN:R00621 R03316 R02570 R07618]
**K00658**	central carbon	TCA	Citrate cycle (TCA cycle, Krebs cycle)	2-oxoglutarate dehydrogenase complex [EC:1.2.4.2 2.3.1.61 1.8.1.4] [RN:R00621 R03316 R02570 R07618]

Table S1. Frequency of genes involved in carbon utilization.

**K01647**	central carbon	TCA	Citrate cycle (TCA cycle, Krebs cycle)	citrate synthase [EC:2.3.3.1] [RN:R00351]
**K01676**	central carbon	TCA	Citrate cycle (TCA cycle, Krebs cycle)	fumarate hydratase [EC:4.2.1.2] [RN:R01082]
**K01679**	central carbon	TCA	Citrate cycle (TCA cycle, Krebs cycle)	fumarate hydratase [EC:4.2.1.2] [RN:R01082]
**K01681**	central carbon	TCA	Citrate cycle (TCA cycle, Krebs cycle)	aconitate hydratase [EC:4.2.1.3] [RN:R01325 R01900 R01324]
**K01682**	central carbon	TCA	Citrate cycle (TCA cycle, Krebs cycle)	aconitate hydratase [EC:4.2.1.3] [RN:R01325 R01900 R01324]
**K01902**	central carbon	TCA	Citrate cycle (TCA cycle, Krebs cycle)	succinyl-CoA synthetase [EC:6.2.1.5] [RN:R00405]
**K01903**	central carbon	TCA	Citrate cycle (TCA cycle, Krebs cycle)	succinyl-CoA synthetase [EC:6.2.1.5] [RN:R00405]
**K00030**	central carbon	TCA	Citrate cycle, first carbon oxidation, oxaloacetate => 2-oxoglutarate	isocitrate dehydrogenase [EC:1.1.1.42 1.1.1.41] [RN:R01899 R00268 R00267 R00709]
**K00031**	central carbon	TCA	Citrate cycle, first carbon oxidation, oxaloacetate => 2-oxoglutarate	isocitrate dehydrogenase [EC:1.1.1.42 1.1.1.41] [RN:R01899 R00268 R00267 R00709]
**K01647**	central carbon	TCA	Citrate cycle, first carbon oxidation, oxaloacetate => 2-oxoglutarate	citrate synthase [EC:2.3.3.1] [RN:R00351]
**K01681**	central carbon	TCA	Citrate cycle, first carbon oxidation, oxaloacetate => 2-oxoglutarate	aconitate hydratase [EC:4.2.1.3] [RN:R01325 R01900 R01324]
**K01682**	central carbon	TCA	Citrate cycle, first carbon oxidation, oxaloacetate => 2-oxoglutarate	aconitate hydratase [EC:4.2.1.3] [RN:R01325 R01900 R01324]
**K00024**	central carbon	TCA	Citrate cycle, second carbon oxidation, 2-	malate dehydrogenase [EC:1.1.1.37] [RN:R00342]

Table S1. Frequency of genes involved in carbon utilization.

			oxoglutarate => oxaloacetate	
**K00116**	central carbon	TCA	Citrate cycle, second carbon oxidation, 2-oxoglutarate => oxaloacetate	malate dehydrogenase (quinone) [EC:1.1.5.4] [RN:R00361]
**K00164**	central carbon	TCA	Citrate cycle, second carbon oxidation, 2-oxoglutarate => oxaloacetate	2-oxoglutarate dehydrogenase [EC:1.2.4.2 2.3.1.61 1.8.1.4] [RN:R00621 R03316 R02570 R07618]
**K00174**	central carbon	TCA	Citrate cycle, second carbon oxidation, 2-oxoglutarate => oxaloacetate	2-oxoglutarate/2-oxoacid ferredoxin oxidoreductase subunit alpha [EC:1.2.7.3 1.2.7.11] [RN:R01197]
**K00175**	central carbon	TCA	Citrate cycle, second carbon oxidation, 2-oxoglutarate => oxaloacetate	2-oxoglutarate/2-oxoacid ferredoxin oxidoreductase subunit beta [EC:1.2.7.3 1.2.7.11] [RN:R01197]
**K00239**	central carbon	TCA	Citrate cycle, second carbon oxidation, 2-oxoglutarate => oxaloacetate	succinate dehydrogenase / fumarate reductase [EC:1.3.5.1 1.3.5.4] [RN:R02164]
**K00240**	central carbon	TCA	Citrate cycle, second carbon oxidation, 2-oxoglutarate => oxaloacetate	succinate dehydrogenase / fumarate reductase [EC:1.3.5.1 1.3.5.4] [RN:R02164]
**K00241**	central carbon	TCA	Citrate cycle, second carbon oxidation, 2-oxoglutarate => oxaloacetate	succinate dehydrogenase / fumarate reductase [EC:1.3.5.1 1.3.5.4] [RN:R02164]
**K00242**	central carbon	TCA	Citrate cycle, second carbon oxidation, 2-oxoglutarate => oxaloacetate	succinate dehydrogenase / fumarate reductase [EC:1.3.5.1 1.3.5.4] [RN:R02164]
**K00244**	central carbon	TCA	Citrate cycle, second carbon oxidation, 2-	fumarate reductase [EC:1.3.5.4] [RN:R02164]

Table S1. Frequency of genes involved in carbon utilization.

			oxoglutarate => oxaloacetate	
**K00382**	central carbon	TCA	Citrate cycle, second carbon oxidation, 2-oxoglutarate => oxaloacetate	2-oxoglutarate dehydrogenase [EC:1.2.4.2 2.3.1.61 1.8.1.4] [RN:R00621 R03316 R02570 R07618]
**K00658**	central carbon	TCA	Citrate cycle, second carbon oxidation, 2-oxoglutarate => oxaloacetate	2-oxoglutarate dehydrogenase [EC:1.2.4.2 2.3.1.61 1.8.1.4] [RN:R00621 R03316 R02570 R07618]
**K01676**	central carbon	TCA	Citrate cycle, second carbon oxidation, 2-oxoglutarate => oxaloacetate	fumarate hydratase [EC:4.2.1.2] [RN:R01082]
**K01679**	central carbon	TCA	Citrate cycle, second carbon oxidation, 2-oxoglutarate => oxaloacetate	fumarate hydratase [EC:4.2.1.2] [RN:R01082]
**K01902**	central carbon	TCA	Citrate cycle, second carbon oxidation, 2-oxoglutarate => oxaloacetate	succinyl-CoA synthetase [EC:6.2.1.5] [RN:R00405]
**K01903**	central carbon	TCA	Citrate cycle, second carbon oxidation, 2-oxoglutarate => oxaloacetate	succinyl-CoA synthetase [EC:6.2.1.5] [RN:R00405]
**K00024**	central carbon	TCA	Incomplete reductive citrate cycle, acetyl-CoA => oxoglutarate	malate dehydrogenase [EC:1.1.1.37] [RN:R00342]
**K00174**	central carbon	TCA	Incomplete reductive citrate cycle, acetyl-CoA => oxoglutarate	2-oxoglutarate ferredoxin oxidoreductase [EC:1.2.7.3] [RN:R01197]
**K00175**	central carbon	TCA	Incomplete reductive citrate cycle, acetyl-CoA => oxoglutarate	2-oxoglutarate ferredoxin oxidoreductase [EC:1.2.7.3] [RN:R01197]

Table S1. Frequency of genes involved in carbon utilization.

**K01902**	central carbon	TCA	Incomplete reductive citrate cycle, acetyl-CoA => oxoglutarate	succinyl-CoA synthetase [EC:6.2.1.5] [RN:R00405]
**K01903**	central carbon	TCA	Incomplete reductive citrate cycle, acetyl-CoA => oxoglutarate	succinyl-CoA synthetase [EC:6.2.1.5] [RN:R00405]
**K01959**	central carbon	TCA	Incomplete reductive citrate cycle, acetyl-CoA => oxoglutarate	pyruvate carboxylase [EC:6.4.1.1] [RN:R00344]
**K01960**	central carbon	TCA	Incomplete reductive citrate cycle, acetyl-CoA => oxoglutarate	pyruvate carboxylase [EC:6.4.1.1] [RN:R00344]
**K00027**	central carbon	TCA	TCA /Reductive TCA	malate dehydrogenase (oxaloacetate-decarboxylating)
**K04117**	hydrocarbon degradation		Benzoate degradation, cyclohexanecarboxylic acid =>pimeloyl-CoA	aliB; cyclohexanecarboxyl-CoA dehydrogenase [EC:1.3.99.-] [RN:R05619]
**K07535**	hydrocarbon degradation		Benzoate degradation, cyclohexanecarboxylic acid =>pimeloyl-CoA	badH; 2-hydroxycyclohexanecarboxyl-CoA dehydrogenase [EC:1.1.1.-] [RN:R05582]
**K00462**	hydrocarbon degradation		Biphenyl degradation, biphenyl => 2-oxopent-4-enoate + benzoate	biphenyl-2,3-diol 1,2-dioxygenase [EC:1.13.11.39] [RN:R03462 R05245]
**K00446**	hydrocarbon degradation		Catechol meta-cleavage, catechol => acetyl-CoA / 4-methylcatechol => propanoyl-CoA	catechol 2,3-dioxygenase [EC:1.13.11.2] [RN:R00816 R05295]
**K01666**	hydrocarbon degradation		Catechol meta-cleavage, catechol => acetyl-CoA / 4-methylcatechol => propanoyl-CoA	4-hydroxy 2-oxovalerate aldolase [EC:4.1.3.39] [RN:R00750]
**K01821**	hydrocarbon degradation		Catechol meta-cleavage, catechol => acetyl-CoA / 4-methylcatechol => propanoyl-CoA	4-oxalocrotonate tautomerase [EC:5.3.2.6] [RN:R03966 R05389]

Table S1. Frequency of genes involved in carbon utilization.

**K02554**	hydrocarbon degradation	Catechol meta-cleavage, catechol => acetyl-CoA / 4-methylcatechol => propanoyl-CoA	2-keto-4-pentenoate hydratase [EC:4.2.1.80] [RN:R02601]
**K04073**	hydrocarbon degradation	Catechol meta-cleavage, catechol => acetyl-CoA / 4-methylcatechol => propanoyl-CoA	acetaldehyde dehydrogenase [EC:1.2.1.10] [RN:R00228]
**K07104**	hydrocarbon degradation	Catechol meta-cleavage, catechol => acetyl-CoA / 4-methylcatechol => propanoyl-CoA	catechol 2,3-dioxygenase [EC:1.13.11.2] [RN:R00816 R05295]
**K10217**	hydrocarbon degradation	Catechol meta-cleavage, catechol => acetyl-CoA / 4-methylcatechol => propanoyl-CoA	2-hydroxymuconate-6-semialdehyde dehydrogenase [EC:1.2.1.85] [RN:R02762 R05353]
**K18364**	hydrocarbon degradation	Catechol meta-cleavage, catechol => acetyl-CoA / 4-methylcatechol => propanoyl-CoA	2-oxopent-4-enoate/cis-2-oxohex-4-enoate hydratase [EC:4.2.1.80 4.2.1.132] [RN:R02601 R05864]
**K18365**	hydrocarbon degradation	Catechol meta-cleavage, catechol => acetyl-CoA / 4-methylcatechol => propanoyl-CoA	4-hydroxy-2-oxovalerate/4-hydroxy-2-oxohexanoate aldolase [EC:4.1.3.39 4.1.3.43] [RN:R00750 R05298]
**K18366**	hydrocarbon degradation	Catechol meta-cleavage, catechol => acetyl-CoA / 4-methylcatechol => propanoyl-CoA	acetaldehyde/propanal dehydrogenase [EC:1.2.1.10 1.2.1.87] [RN:R00228 R09097]
**K01055**	hydrocarbon degradation	Catechol ortho-cleavage, catechol => 3-oxoadipate	3-oxoadipate enol-lactonase [EC:3.1.1.24] [RN:R02991]
**K01856**	hydrocarbon degradation	Catechol ortho-cleavage, catechol => 3-oxoadipate	muconate cycloisomerase [EC:5.5.1.1] [RN:R06989]

Table S1. Frequency of genes involved in carbon utilization.

**K03381**	hydrocarbon degradation	Catechol ortho-cleavage, catechol => 3-oxoadipate	catechol 1,2-dioxygenase [EC:1.13.11.1] [RN:R00817]
**K18106**	hydrocarbon degradation	D-Galacturonate degradation (fungi), D-galacturonate => glycerol	D-galacturonate reductase [EC:1.1.1.-] [RN:R07676 R10565]
**K00151**	hydrocarbon degradation	Homoprotocatechuate degradation, homoprotocatechuate => 2-oxohept-3-enedioate	5-carboxymethyl-2-hydroxymuconic-semialdehyde dehydrogenase [EC:1.2.1.60] [RN:R04418]
**K01826**	hydrocarbon degradation	Homoprotocatechuate degradation, homoprotocatechuate => 2-oxohept-3-enedioate	5-carboxymethyl-2-hydroxymuconate isomerase [EC:5.3.3.10][RN:R04379]
**K05921**	hydrocarbon degradation	Homoprotocatechuate degradation, homoprotocatechuate => 2-oxohept-3-enedioate	5-oxopent-3-ene-1,2,5-tricarboxylate decarboxylase / 2-hydroxyhepta-2,4-diene-1,7-dioate isomerase [EC:4.1.1.68 5.3.3.-] [RN:R04380 R04134]
**K14581**	hydrocarbon degradation	Naphthalene degradation, naphthalene => salicylate	nahA; naphthalene 1,2-dioxygenase [EC:1.14.12.12] [RN:R02968]
**K14584**	hydrocarbon degradation	Naphthalene degradation, naphthalene => salicylate	2-hydroxychromene-2-carboxylate isomerase [EC:5.99.1.4] [RN:R05137]
**K04102**	hydrocarbon degradation	Phthalate degradation, phthalate => protocatechuate	4,5-dihydroxyphthalate decarboxylase [EC:4.1.1.55] [RN:R01635]
**K00448**	hydrocarbon degradation	protocatechuate degradation,	pcaG; protocatechuate 3,4-dioxygenase, alpha subunit [EC:1.13.11.3]

Table S1. Frequency of genes involved in carbon utilization.

		protocatechuate => 3-oxoadipate	
**K00449**	hydrocarbon degradation	protocatechuate degradation, protocatechuate => 3-oxoadipate	pcaH; protocatechuate 3,4-dioxygenase, beta subunit [EC:1.13.11.3]
**K01607**	hydrocarbon degradation	protocatechuate degradation, protocatechuate => 3-oxoadipate	pcaC; 4-carboxymuconolactone decarboxylase [EC:4.1.1.44]
**K01857**	hydrocarbon degradation	protocatechuate degradation, protocatechuate => 3-oxoadipate	pcaB; 3-carboxy-cis,cis-muconate cycloisomerase [EC:5.5.1.2]
**K10218**	hydrocarbon degradation	protocatechuate degradation, protocatechuate => oxaloacteate + pyruvate	ligK; 4-hydroxy-4-methyl-2-oxoglutarate aldolase [EC:4.1.3.17]
**K16514**	hydrocarbon degradation	protocatechuate degradation, protocatechuate => oxaloacteate + pyruvate	galD; 4-oxalomesaconate tautomerase [EC:5.3.2.8]
**K00529**	hydrocarbon degradation	Trans-cinnamate degradation, trans-cinnamate => acetyl-CoA	3-phenylpropionate/cinnamic acid dioxygenase [EC:1.14.12.19] [RN:R06783]
**K01666**	hydrocarbon degradation	Trans-cinnamate degradation, trans-cinnamate => acetyl-CoA	4-hydroxy 2-oxovalerate aldolase [EC:4.1.3.39] [RN:R00750]
**K02554**	hydrocarbon degradation	Trans-cinnamate degradation, trans-cinnamate => acetyl-CoA	2-keto-4-pentenoate hydratase [EC:4.2.1.80] [RN:R02601]
**K04073**	hydrocarbon degradation	Trans-cinnamate degradation, trans-	acetaldehyde dehydrogenase [EC:1.2.1.10] [RN:R00228]

Table S1. Frequency of genes involved in carbon utilization.

		cinnamate => acetyl-CoA	
**K05710**	hydrocarbon degradation	Trans-cinnamate degradation, trans-cinnamate => acetyl-CoA	3-phenylpropionate/cinnamic acid dioxygenase [EC:1.14.12.19] [RN:R06783]
**K05712**	hydrocarbon degradation	Trans-cinnamate degradation, trans-cinnamate => acetyl-CoA	mhpA; 3-(3-hydroxy-phenyl)propionate hydroxylase [EC:1.14.13.127] [RN:R06787]
**K05714**	hydrocarbon degradation	Trans-cinnamate degradation, trans-cinnamate => acetyl-CoA	2-hydroxy-6-ketonona-2,4-dienedioic acid hydrolase [EC:3.7.1.14] [RN:R06789]
**K00016**	pyruvate metabolism	fermentation-	L-lactate dehydrogenase
**K03778**	pyruvate metabolism	fermentation-	D-lactate dehydrogenase
**K00625**	pyruvate metabolism	Phosphate acetyltransferase-acetate kinase pathway, acetyl-CoA => acetate	phosphate acetyltransferase [EC:2.3.1.8] [RN:R00230]
**K00925**	pyruvate metabolism	Phosphate acetyltransferase-acetate kinase pathway, acetyl-CoA => acetate	acetate kinase [EC:2.7.2.1] [RN:R00315]
**K13788**	pyruvate metabolism	Phosphate acetyltransferase-acetate kinase pathway, acetyl-CoA => acetate	phosphate acetyltransferase [EC:2.3.1.8] [RN:R00230]
**K01847**	pyruvate metabolism	Propanoyl-CoA metabolism, propanoyl-CoA => succinyl-CoA	methylmalonyl-CoA mutase [EC:5.4.99.2] [RN:R00833]
**K01849**	pyruvate metabolism	Propanoyl-CoA metabolism, propanoyl-CoA => succinyl-CoA	methylmalonyl-CoA mutase [EC:5.4.99.2] [RN:R00833]

Table S1. Frequency of genes involved in carbon utilization.

**K01965**	pyruvate metabolism		Propanoyl-CoA metabolism, propanoyl-CoA => succinyl-CoA	propionyl-CoA carboxylase [EC:6.4.1.3] [RN:R01859]
**K01966**	pyruvate metabolism		Propanoyl-CoA metabolism, propanoyl-CoA => succinyl-CoA	propionyl-CoA carboxylase [EC:6.4.1.3] [RN:R01859]
**K05606**	pyruvate metabolism		Propanoyl-CoA metabolism, propanoyl-CoA => succinyl-CoA	methylmalonyl-CoA/ethylmalonyl-CoA epimerase [EC:5.1.99.1] [RN:R02765]
**K00074**	pyruvate metabolism		pyruvate metabolism	3-hydroxybutyryl-CoA dehydrogenase
**K01512**	pyruvate metabolism		pyruvate metabolism	acylphosphatase
**K00161**	pyruvate metabolism		Pyruvate oxidation, pyruvate => acetyl-CoA	pyruvate dehydrogenase complex [EC:1.2.4.1 2.3.1.12 1.8.1.4] [RN:R00209]
**K00162**	pyruvate metabolism		Pyruvate oxidation, pyruvate => acetyl-CoA	pyruvate dehydrogenase complex [EC:1.2.4.1 2.3.1.12 1.8.1.4] [RN:R00209]
**K00163**	pyruvate metabolism		Pyruvate oxidation, pyruvate => acetyl-CoA	pyruvate dehydrogenase complex [EC:1.2.4.1 2.3.1.12 1.8.1.4] [RN:R00209]
**K00382**	pyruvate metabolism		Pyruvate oxidation, pyruvate => acetyl-CoA	pyruvate dehydrogenase complex [EC:1.2.4.1 2.3.1.12 1.8.1.4] [RN:R00209]
**K00627**	pyruvate metabolism		Pyruvate oxidation, pyruvate => acetyl-CoA	pyruvate dehydrogenase complex [EC:1.2.4.1 2.3.1.12 1.8.1.4] [RN:R00209]
**K03737**	pyruvate metabolism		Pyruvate oxidation, pyruvate => acetyl-CoA	pyruvate:ferredoxin oxidoreductase [EC:1.2.7.1] [RN:R01196]
**K01812**	sugar utilization (woodcroft)	galacturonic acid degradation	Pentose and glucuronate interconversions	glucuronate isomerase
**K05810**		Polyphenolics Cleavage	polyphenols	polyphenol oxidase
