# Supplementary material for: Extracellular Polymers from Nitzschia sp. for Removing Clay Minerals from Water in Mining
Source: Polymers (Basel). 2026 May 17;18(10):1221. doi: 10.3390/polym18101221 (PMC13210723; doi:10.3390/polym18101221)
Supplement: Supplementary file 1 [file polymers-18-01221-s001.zip › Table S2, S3 Figure S1, S2 revMR.pdf]

Table S2. Factors and levels for DBB.

Factors	*Level min*	*Level mid*	*Level max*
	–1	0	1
Na_2CO_3 gL^{-1}	0.02	0.21	0.4
pH initial	6	8	10
Na_2SiO_3 gL^{-1}	0.1	0.3	0.5

Table S3. Resulting combinations correspond to the different growing conditions.

Condition	*Na_2CO_3 gL^{-1}*	*pH*	*Na_2SiO_3 gL^{-1}*
1	0.02	8	0.1
2	0.21	6	0.1
3	0.02	10	0.3
4	0.21	8	0.3
5	0.4	8	0.1
6	0.4	10	0.3
7	0.4	6	0.3
8	0.02	8	0.5
9	0.21	10	0.1
10	0.21	10	0.5
11	0.21	6	0.5
12	0.4	8	0.5
13	0.21	8	0.3
14	0.02	6	0.3
15	0.21	8	0.3

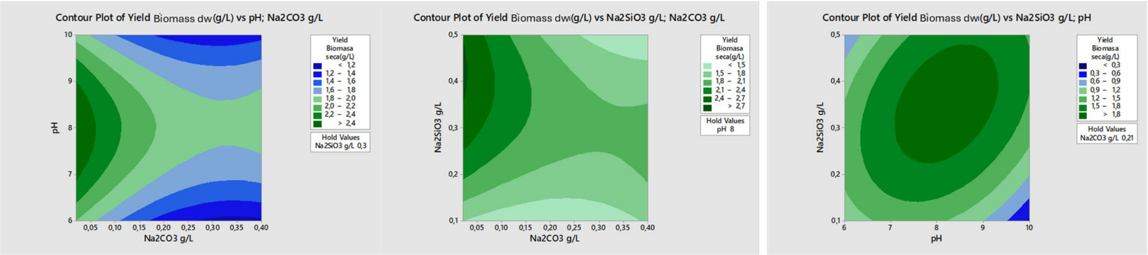

Figure S1. Response surface plots, biomass analysis.

Response Surface Regression: Yield Biomasa seca(g/L) versus Na2CO3 g/L; pH; Na2SiO3 g/L

Analysis of Variance

Source	DF	Adj SS	Adj MS	F-Value	P-Value
Model	9	5,24464	0,58274	12,50	0,006
Linear	3	1,36508	0,45503	9,76	0,016
Na2CO3 g/L	1	0,99053	0,99053	21,25	0,006
pH	1	0,00475	0,00475	0,10	0,762
Na2SiO3 g/L	1	0,36980	0,36980	7,93	0,037
Square	3	2,67585	0,89195	19,14	0,004
Na2CO3 g/L*Na2CO3 g/L	1	0,29467	0,29467	6,32	0,054
pH*pH	1	1,46548	1,46548	31,45	0,002
Na2SiO3 g/L*Na2SiO3 g/L	1	0,88201	0,88201	18,93	0,007
2-Way Interaction	3	1,20371	0,40124	8,61	0,020
Na2CO3 g/L*pH	1	0,01690	0,01690	0,36	0,573
Na2CO3 g/L*Na2SiO3 g/L	1	0,55876	0,55876	11,99	0,018
pH*Na2SiO3 g/L	1	0,62806	0,62806	13,48	0,014
Error	5	0,23302	0,04660		
Lack-of-Fit	3	0,15437	0,05146	1,31	0,461
Pure Error	2	0,07865	0,03932		
Total	14	5,47766			

Model Summary

S	R-sq	R-sq(adj)	R-sq(pred)
0,215879	95,75%	88,09%	51,68%

Coded Coefficients

Term	Effect	Coef	SE Coef	T-Value	P-Value	VIF
Constant		1,945	0,125	15,61	0,000	
Na2CO3 g/L	-0,7038	-0,3519	0,0763	-4,61	0,006	1,00
pH	0,0487	0,0244	0,0763	0,32	0,762	1,00
Na2SiO3 g/L	0,4300	0,2150	0,0763	2,82	0,037	1,00
Na2CO3 g/L*Na2CO3 g/L	0,565	0,282	0,112	2,51	0,054	1,01
pH*pH	-1,260	-0,630	0,112	-5,61	0,002	1,01
Na2SiO3 g/L*Na2SiO3 g/L	-0,978	-0,489	0,112	-4,35	0,007	1,01
Na2CO3 g/L*pH	0,130	0,065	0,108	0,60	0,573	1,00
Na2CO3 g/L*Na2SiO3 g/L	-0,747	-0,374	0,108	-3,46	0,018	1,00
pH*Na2SiO3 g/L	0,792	0,396	0,108	3,67	0,014	1,00

Regression Equation in Uncoded Units

```
Yield Biomasa seca(g/L) = -6,88 - 3,56 Na2CO3 g/L + 2,199 pH + 2,55 Na2SiO3 g/L
                          + 7,83 Na2CO3 g/L*Na2CO3 g/L - 0,1575 pH*pH
                          - 12,22 Na2SiO3 g/L*Na2SiO3 g/L + 0,171 Na2CO3 g/L*pH
                          - 9,84 Na2CO3 g/L*Na2SiO3 g/L + 0,991 pH*Na2SiO3 g/L
```

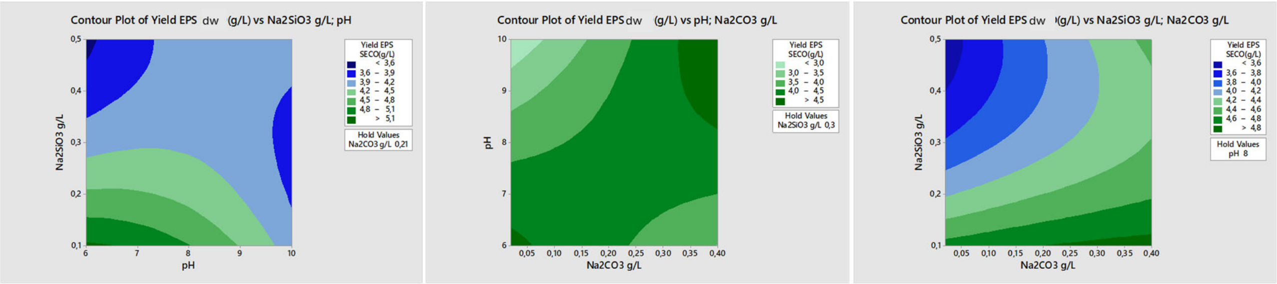

Figure S2. Response surface plots, EPS dry weight analysis.

Response Surface Regression: Yield EPS SECO(g/L) versus Na2CO3 g/L; pH; Na2SiO3 g/L

Analysis of Variance

Source	DF	Adj SS	Adj MS	F-Value	P-Value
Model	9	6,29909	0,69990	3,58	0,087
Linear	3	2,07256	0,69085	3,54	0,104
Na2CO3 g/L	1	0,68404	0,68404	3,50	0,120
pH	1	0,14426	0,14426	0,74	0,429
Na2SiO3 g/L	1	1,24425	1,24425	6,37	0,053
Square	3	0,46160	0,15387	0,79	0,550
Na2CO3 g/L*Na2CO3 g/L	1	0,00383	0,00383	0,02	0,894
pH*pH	1	0,16044	0,16044	0,82	0,406
Na2SiO3 g/L*Na2SiO3 g/L	1	0,26198	0,26198	1,34	0,299
2-Way Interaction	3	3,76493	1,25498	6,42	0,036
Na2CO3 g/L*pH	1	2,92290	2,92290	14,96	0,012
Na2CO3 g/L*Na2SiO3 g/L	1	0,14063	0,14063	0,72	0,435
pH*Na2SiO3 g/L	1	0,70141	0,70141	3,59	0,117
Error	5	0,97711	0,19542		
Lack-of-Fit	3	0,76260	0,25420	2,37	0,311
Pure Error	2	0,21452	0,10726		
Total	14	7,27620			

Model Summary

S	R-sq	R-sq(adj)	R-sq(pred)
0,442066	86,57%	62,40%	0,00%

Coded Coefficients

Term	Effect	Coef	SE Coef	T-Value	P-Value	VIF
Constant		4,148	0,255	16,25	0,000	
Na2CO3 g/L	0,585	0,292	0,156	1,87	0,120	1,00
pH	-0,269	-0,134	0,156	-0,86	0,429	1,00
Na2SiO3 g/L	-0,789	-0,394	0,156	-2,52	0,053	1,00
Na2CO3 g/L*Na2CO3 g/L	-0,064	-0,032	0,230	-0,14	0,894	1,01
pH*pH	-0,417	-0,208	0,230	-0,91	0,406	1,01
Na2SiO3 g/L*Na2SiO3 g/L	0,533	0,266	0,230	1,16	0,299	1,01
Na2CO3 g/L*pH	1,710	0,855	0,221	3,87	0,012	1,00
Na2CO3 g/L*Na2SiO3 g/L	0,375	0,188	0,221	0,85	0,435	1,00
pH*Na2SiO3 g/L	0,838	0,419	0,221	1,89	0,117	1,00

Regression Equation in Uncoded Units

```
Yield EPS SECO(g/L) = 8,78 - 17,56 Na2CO3 g/L - 0,020 pH - 15,38 Na2SiO3 g/L
                      - 0,89 Na2CO3 g/L*Na2CO3 g/L - 0,0521 pH*pH
                      + 6,66 Na2SiO3 g/L*Na2SiO3 g/L + 2,250 Na2CO3 g/L*pH
                      + 4,93 Na2CO3 g/L*Na2SiO3 g/L + 1,047 pH*Na2SiO3 g/L
```
