# Supplementary material for: Extracellular Polymers from Nitzschia sp. for Removing Clay Minerals from Water in Mining
Source: Polymers (Basel). 2026 May 17;18(10):1221. doi: 10.3390/polym18101221 (PMC13210723; doi:10.3390/polym18101221)
Supplement: Supplementary file 1 [file polymers-18-01221-s001.zip › Table S4 revMR.pdf]

Table S4. EPS productivity (mg·L^{-1}·d^{-1}) of *Nitzschia* sp. strain 53.3 under different sodium metasilicate, glucose, and sodium carbonate treatments. Data corresponds to measurements taken on days 7 and 17.

**Treatments**	**EPS productivity mgL^{-1}d^{-1}**		
	Concentrations	Day 7	Day 17
**Control**	0	114.3 ±4.37	14 ±3.4
**Sodium metasilicate**	0.03	99.3 ±3.1	57.5 ±7.5
**Na_2SiO_3 gL^{-1}**	0.30	144.8 ±10.79	52.9 ±9
	1.00	113.7 ±6.05	4.1 ±1.05
	1.50	156.0 ±4.16	63.1 ±1.04
	2.00	98.1 ±6.51	16.5 ±3.91
	2.50	120.0 ±7.42	3.5 ±1.77
**Glucose $C_6H_{12}O_6$ gL^{-1}**	0.10	132.6 ±3.38	75.6 ±5.89
	1.00	120.2 ±4.25	58.9 ±5.47
	1.50	32.4 ±1.11	7.8 ±1.45
	2.00	29.2 ±2.82	12.4 ±2.54
	2.50	51.1 ±2.65	31.6 ±2.5
**Sodium carbonate**	0.02	143.6 ±2.23	26.0 ±2.12
**Na_2CO_3 gL^{-1}**	0.20	139.3 ±1.83	25.8 ±1.2
	1.00	18.8 ±3.28	8.2 ±2.62
	1.50	5.7 ±1.76	21.8 ±1.21
	2.00	29.2 ±3.28	14.2 ±2.62
	2.50	51.1 ±1.76	11.1 ±1.21
